# The Chp1 chromodomain binds the H3K9me tail and the nucleosome core to assemble heterochromatin

**DOI:** 10.1038/celldisc.2016.4

**Published:** 2016-04-19

**Authors:** Manuel Zocco, Mirela Marasovic, Paola Pisacane, Silvija Bilokapic, Mario Halic

**Affiliations:** 1 Department of Biochemistry, Gene Center, University of Munich, Munich, Germany

**Keywords:** Chp 1, chromodomain, cryo-EM, heterochromatin, nucleosome, RNAi, *S. pombe*

## Abstract

To maintain genome stability, cells pack large portions of their genome into silent chromatin or heterochromatin. Histone H3 lysine 9 methylation, a hallmark of heterochromatin, is recognized by conserved readers called chromodomains. But how chromodomains interact with their actual binding partner, the H3K9 methylated nucleosome, remains elusive. We have determined the structure of a nucleosome trimethylated at lysine 9 of histone H3 (H3K9me3 Nucleosome) in a complex with the chromodomain of Chp1, a protein required for RNA interference-dependent heterochromatin formation in fission yeast. The cryo-electron microscopy structure reveals that the chromodomain of Chp1 binds the histone H3 lysine 9 methylated tail and the core of the nucleosome, primarily histones H3 and H2B. Mutations in chromodomain of Chp1 loops, which interact with the nucleosome core, abolished this interaction *in vitro.* Moreover, fission yeast cells with Chp1 loop mutations have a defect in Chp1 recruitment and heterochromatin formation. This study reveals the structural basis for heterochromatic silencing and suggests that chromodomains could read histone code in the H3 tail and the nucleosome core, which would provide an additional layer of regulation.

## Introduction

To maintain genome stability, cells pack large portions of their genome into silent chromatin or heterochromatin. Heterochromatin is required for centromere formation, gene silencing, repression of recombination and maintenance of telomere stability. A class of histone methyltransferases, the Su(var) family, specifically deposits the histone H3 lysine K9 methylation (H3K9me), which is recognized by the chromodomain (CD)-containing Heterochromatin Protein 1 (HP1) family of proteins. HP1 proteins bind to H3K9me nucleosomes and mediate chromatin compaction and transcriptional gene silencing [1–4]. Mutations in chromodomains disrupt the HP1 localization to heterochromatin, indicating that the chromodomain has a primary role in HP1 targeting to chromatin [5].

In fission yeast small RNAs guide the RNA induced transcriptional silencing (RITS) complex to centromeric repeats to induce H3K9 methylation and heterochromatin formation [6]. In the RITS complex, Argonaute interacts with two additional proteins, Tas3 and Chp1. Tas3, a glycine and tryptophan-motif-containing protein, links Argonaute to Chp1 [7, 8]. Chp1, a chromodomain-containing protein, is essential for heterochromatin formation in fission yeast and specifically interacts with H3K9-methylated Nucleosomes [9–11]. RITS can therefore associate with chromatin through base-pairing interactions of siRNAs in Argonaute with nascent transcripts and binding of Chp1 to H3K9-methylated Nucleosomes [6]. This leads to the recruitment of the Su(var) family Clr4 methyltransferase complex to chromatin, additional cycles of H3K9 methylation, and recruitment of two other chromodomain proteins, Swi6 and Chp2, the fission yeast HP1 homologs [12–14].

In *Schizosaccharomyces pombe,* four chromodomain proteins are involved in heterochromatin formation and transcriptional gene silencing: Chp1, Chp2, Clr4 and Swi6. The chromodomain of Chp1 (Chp1CD) has the highest affinity for the H3K9me peptide and is essential for tethering the RITS complex to centromeric region and for heterochromatin establishment [10, 15]. Chp1CD and several other chromodomains can bind DNA or RNA as well [16–18]. This intrinsic nucleic acid-binding activity of Chp1CD is required for heterochromatin formation in fission yeast [17]. The chromodomain of Clr4 links the deposition of H3K9 methylation with the readout and provides a feed-forward mechanism for amplification and spreading of the initially deposited mark [19]. The chromodomains of the HP1 proteins, Swi6 and Chp2, bind the methylated H3K9 to induce a silent chromatin structure through largely non-overlapping inhibitory mechanisms [1, 3, 20, 21]. Different affinities of chromodomains for H3K9me2/3 and neighboring H3K4 acetylation mark can also contribute to their distinct function in the establishment of H3K9me and the spreading of heterochromatin [22, 23].

The structures of multiple chromodomains bound to H3K9me peptides have been solved by NMR spectrometry and X-ray crystallography [10, 17, 24, 25]. The chromodomain consists of three β-strands and an α-helix and recognizes the H3K9me tail through an aromatic cage. Despite multiple structures of isolated chromodomains bound to H3K9me peptides [10, 24, 25], it remains unclear how chromodomains interact with their actual binding partner, the H3K9 methylated nucleosome. This interaction determines how chromodomains can coordinate the different functions of the above mentioned proteins at the same locus.

We have solved the structure of a chromodomain (Chp1CD) bound to a H3K9me3Nucleosome by cryo-electron microscopy (cryo-EM). Contrary to expectations, Chp1CD interacts not only with the H3K9me tail, but it also makes contacts with the core of the nucleosome, primarily with histone H3. The loops of Chp1CD bind the core of the nucleosome, whereas the positively charged α-helix is oriented outwards and could indeed tether nascent RNAs as suggested [17]. We mutated residues in two loops that interact with the nucleosome core and show that, although Chp1CD specifically recognizes H3K9me, the tethering to the core further stabilizes the complex *in vitro*. The same mutations abolished Chp1 recruitment to centromeric repeats and their silencing in fission yeast cells. Our data show that interaction of chromodomains with the nucleosome is far more complex than just binding to the histone tails and suggest that chromodomains could read histone code in the nucleosome core that would provide an additional layer of regulation.

## Results

### Structure of Chp1CD-H3K9me3Nucleosome complex

**Figure 1:**
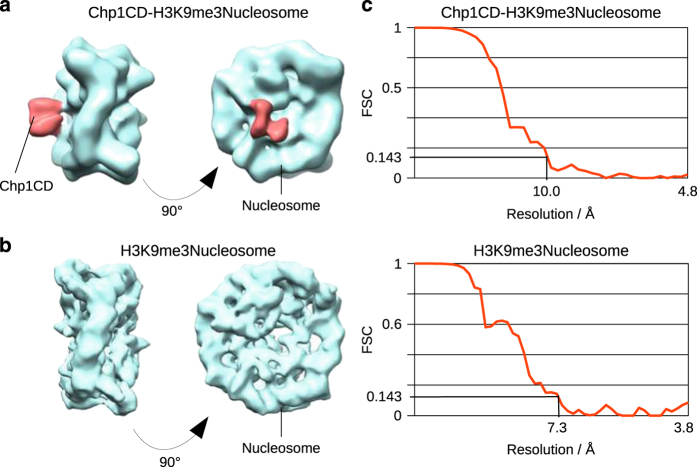
Cryo-EM reconstruction of Chp1CD-H3K9me3Nucleosome complex. (**a**) Cryo-EM map of Chp1CD-H3K9me3Nucleosome complex at 10 Å (FSC 0.143 cutoff of two independently refined data sets). The map was reconstructed from the C15 subclass that had defined electron density in addition to the core of the nucleosome (depicted in red). The nucleosome is shown in light blue. No symmetry was applied. (**b**) Cryo-EM map of the H3K9me3Nucleosome control at 7.3 Å (FSC 0.143 cutoff of two independently refined data sets). The map shows the core of the nucleosome (light blue). No density is found at the position of Chp1CD density in the Chp1CD-H3K9me3Nucleosome complex cryo-EM map. C2 symmetry was applied. (**c**) Fourier shell correlation (FSC) curve showing the resolution of cryo-EM maps shown in **a** and **b**. The resolution is shown at 0.143 cutoff for both reconstructions. The mask including nucleosome and the Chp1 density was applied for resolution calculation for both density maps. Both data sets were split into two halves that were independently refined.

To investigate this interaction we assembled H3K9me3Nucleosomes *in vitro* as previously described [26, 27] (Supplementary Figure S1A–E). H3K9me3 Nucleosomes were bound to resin-associated Chp1CD, the complex was eluted and used in negative stain and cryo-EM (Supplementary Figure S1F–I). We have reconstructed cryo-EM map of the Chp1CD-H3K9me3Nucleosome complex (class C15) at 10 Å resolution using C1 symmetry. For the control map of H3K9me3Nucleosome, we used C2 symmetry and reached resolution of 7.3 Å (Figure 1a–c).

Chp1CD-H3K9me3Nucleosome complex single-particle images were classified to enrich for the ligand occupancy generating initial classes C1–C6. The classified cryo-EM map of the Chp1CD-H3K9me3Nucleosome complex (C1) showed a prominent density in addition to the core nucleosome particle (Supplementary Figure S2A and B). Classification of control H3K9me3Nucleosome particles (classes N1–N5) did not lead to the appearance of defined density outside the nucleosome core indicating that the additional density in the Chp1CD-H3K9me3Nucleosome map is generated by the associated chromodomain (Figure 1b; Supplementary Figure S2C). The small undefined density observed in N1 class of the H3K9me3Nucleosome particles could be generated either by histone tails or by noise and does not overlap with the Chp1CD (Supplementary Figure S2D).

The additional density in the C1 class of Chp1CD-H3K9me3Nucleosome complex was featureless and, at low contour level, larger than Chp1CD is expected to be. This suggested a likely mixture of multiple conformations in our electron density map. Using unsupervised 3D classification of single-particle images we have further classified the complex and generated five cryo-EM maps (C11–C15) of the Chp1CD-H3K9me3Nucleosome complex (Supplementary Figure S3A). Two classes C11 and C12, comprising 60% of particles, contain mainly empty nucleosomes and have only very weak additional density that is only visible at low contour levels. The remaining three classes containing 61 000 particles show clear density in addition to the nucleosome core. In two classes, C13 and C14, we find less-defined and featureless additional densities. In these classes, the putative Chp1CD density is larger than Chp1CD should be suggesting additional contribution to this density next to Chp1CD (Supplementary Figure S3A). The additional density might come from histone tails, SUMO tag or from noise. Local resolution calculated with the Resolution Map software (ResMap) [28] of putative Chp1CD densities in classes C13 and C14 is above 15 Å, making these classes uninterpretable.

**Figure 2:**
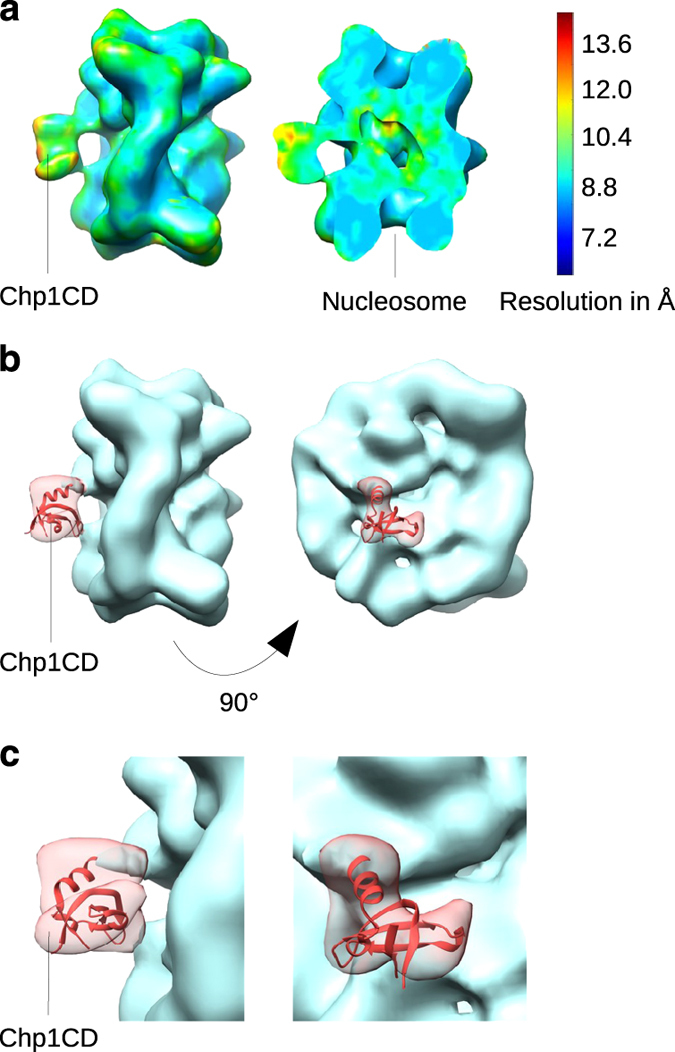
Model of Chp1CD bound to the nucleosome core. (**a**) Local resolution for Chp1CD-H3K9me3Nucleosome Cryo-EM map was calculated with the Resolution Map software (ResMap). The local resolution calculation shows that nucleosome core resolution is 9–10 Å, whereas Chp1CD has a slightly lower resolution of 10 Å. (**b** and **c**) Fitting of Chp1CD (PDB code 3G7L, red) crystal structure into the Chp1CD-H3K9me3Nucleosome Cryo-EM map (cross-correlation 0.91). Note the separation of Chp1CD density into two features where α-helix and β-sheet were fitted in the cryo-EM map (transparent red). The nucleosome is shown in light blue.

In class C15, comprising 55% of the particles having additional density (C13–C15), the Chp1CD density is smaller, much more defined, and shows strong interaction with the core of the nucleosome (Figure 1a; Supplementary Figure S3A). As SUMO does not interact with the nucleosome (Supplementary Figure S1H), this density can only be generated by Chp1CD. Local resolution calculation shows that in the C15 cryo-EM map the resolution of the nucleosomal core is between 9 and 10 Å, whereas the slightly less-defined Chp1CD density has local resolution of 10 Å (Figure 2a). This indicates a high enrichment for one distinct conformation of Chp1CD bound to the nucleosome core. Altogether, more than half of particles containing any additional density in addition to the nucleosome density show strong connection to the nucleosome core (Figure 1a; Supplementary Figure 3A). To confirm Chp1CD binding to nucleosome core, we have used an antibody against SUMO tagged Chp1CD protein. In negative stain images, we find that the antibody localized to the nucleosome core at the place similar to where Chp1CD binds in our cryo-EM maps (Supplementary Figure S3B). Our cryo-EM data show that Chp1CD binds both the H3K9me tail and the core of the nucleosome.

### Chp1CD binds the core of the nucleosome

We fitted the crystal structure of the nucleosome (PDB code 3LZ0) [29] into the density maps of the Chp1CD-H3K9me3Nucleosome complex and the H3K9me3Nucleosome control (Supplementary Figure S4A). The DNA as well as many α-helices of histones are clearly resolved in the cryo-EM maps allowing precise fitting of the nucleosome crystal structure (Supplementary Figure S4A and B). The features present in the cryo-EM maps show that maps are indeed resolved at 7.3 Å (H3K9me3 Nucleosome) and 10 Å (Chp1CD-H3K9me3Nucleosome, C15). In both, Chp1CD-H3K9me3Nucleosome and H3K9me3 Nucleosome reconstructions, we find that the DNA at entry/exit sites is more flexible. At high contour level, the density comprising first and last seven base pairs is barely visible, whereas at low contour level this density is bigger and less defined, suggesting that the last few nucleotides of DNA are more loosely bound to the histone octamer (Supplementary Figure S4C).

In Chp1CD-H3K9me3Nucleosome map, the Chp1CD density had a defined shape and features that allowed fitting of the crystal structure of Chp1CD (PDB code 3G7L) (Figure 2b) [10]. Although we cannot build a very precise model of Chp1CD for such a small and possibly dynamic complex, by combining the information from cryo-EM maps and mutational analysis (see later), we can reliably define the interaction interface between Chp1CD and the nucleosome. In our structure we can see a clear separation of the Chp1CD cryo-EM map into two distinct features in which we have fitted α-helix and β-sheet of Chp1CD. We fitted the crystal structure of the Chp1CD into the Chp1CD-H3K9me3Nucleosome cryo-EM map with its β-sheet facing the core of the nucleosome and the α-helix pointing toward H2B (cross-correlation 0.91) (Figure 2b and c).

**Figure 3:**
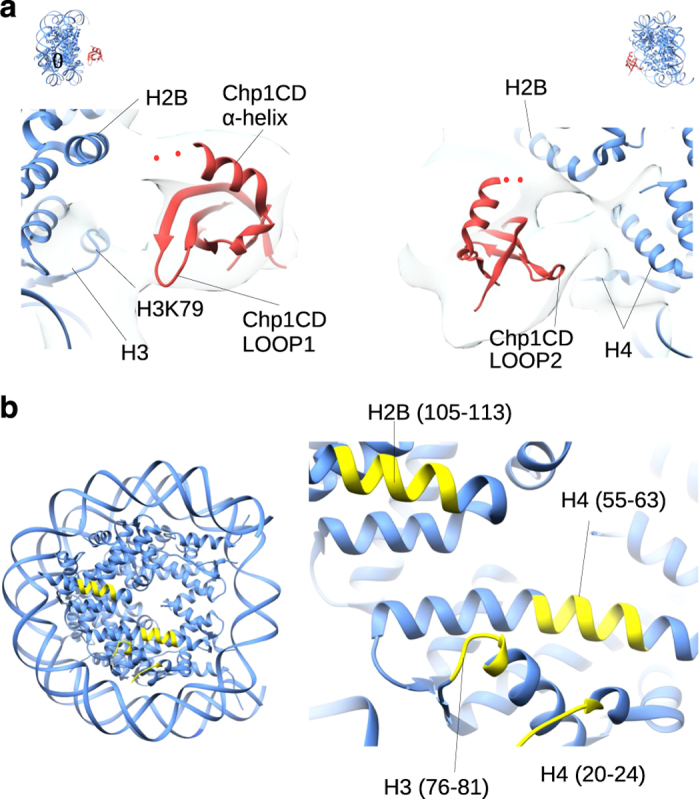
Interaction of Chp1CD with the nucleosome core. (**a**) Molecular interface of Chp1CD interaction with the H3K9me3Nucleosome. Three contacts can be observed. Chp1CD LOOP1 (31–37aa) interacts with the histone H3 loop (77–81aa), whereas Chp1CD LOOP2 interacts with H4 (55–63aa) and potentially also with H4 tail. Chp1CD α-helix interacts with acidic patch formed by H2B (105–113aa). Chp1CD is shown in red and the nucleosome in blue. The cryo-EM map is shown in transparent light blue. Red dots represent couple of residues present in the protein but absent in the crystal structure. (**b**) Depiction of the nucleosomal regions that make contacts with Chp1CD (colored yellow). Primary interaction is with histone H3 region 76–81aa. Second observed interaction is with an acidic patch formed by H2A and H2B (H2B, region 105–113aa). At lower contour level we observe an interaction with H4 region 55–63aa and possibly with H4 tail.

In Chp1CD-H3K9me3Nucleosome cryo-EM map we observe three contacts between Chp1CD and the nucleosome core. These contacts are formed by the two loops of Chp1CD β-sheet and by the tip of the α-helix (Figure 2c). Chp1CD LOOP1 (region 30–38aa) makes a contact with the histone H3 loop (region 76–81aa), whereas the second loop of Chp1CD β-sheet (region 48–52aa) makes contacts with an α-helix of H4 (region 53–65aa) and possibly with H4 tail (Figure 3a and b). The third contact is made by the tip of the positively charged α-helix, which contacts the acidic patch of the nucleosome formed by histones H2A and H2B. It should be considered that several residues at the C-terminus of α-helix are missing in the crystal structures of Chp1CD but are present in our protein. In our model, most lysines in the α-helix point outwards and might bind an RNA as previously suggested (Figure 3a and b) [17]. Our structure shows that Chp1CD binds the nucleosome solely through protein–protein interactions with histones and makes multiple contacts with the nucleosome core.

We can also trace a density stretching from Chp1CD density to the DNA and then along the DNA to the site where H3 tail exits the core of the nucleosome (Supplementary Figure S4D). The density goes only a very short way along the histone surface to the DNA and then along the DNA helix to the site where H3 tail exits the nucleosome core in crystal structures. The density is not very defined and only visible at lower contour levels as expected for an unstructured histone tail. Furthermore, our finding suggests that H3 tail might adopt a preferred conformation when bound to Chp1CD and goes along the negatively charged DNA to the Chp1CD.

### Chp1CD binding to the core is required for Chp1CD-H3K9me3Nucleosome complex formation

**Figure 4:**
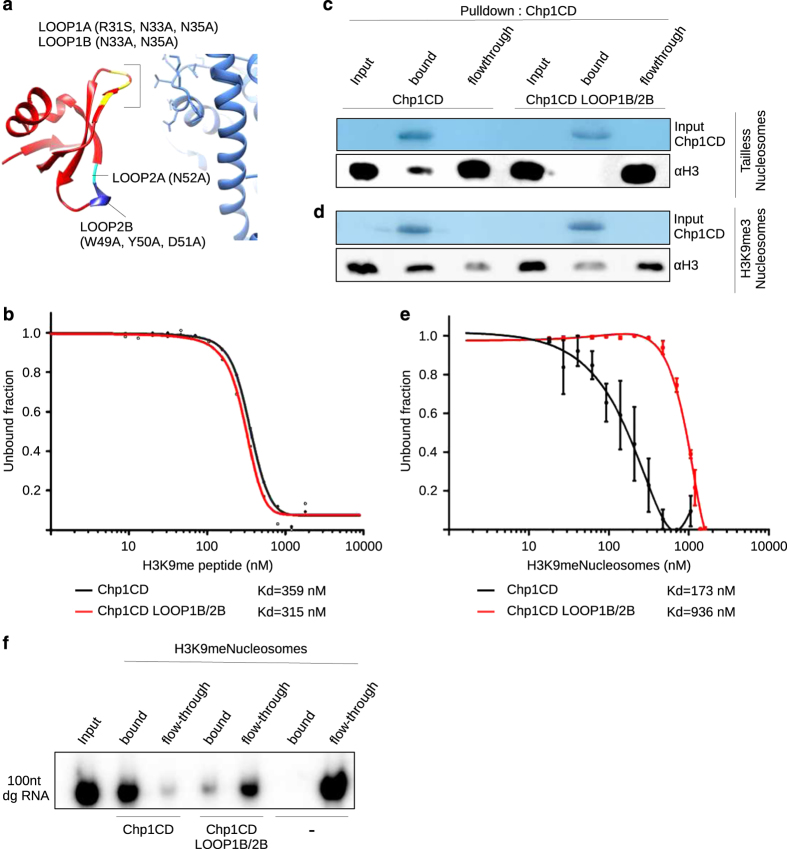
Chp1CD interaction with the nucleosome core is required for binding to H3K9me3Nucleosomes. (**a**) Model of Chp1CD interacting with the H3K9me3Nucleosome. Mutations used in this study are depicted on the model. LOOP1A/B mutations are shown in yellow, LOOP2A in cyan, LOOP2B in blue. (**b**) Thermophoresis assay showing binding curves of wild-type Chp1CD and LOOP1B/2B Chp1CD mutant to H3K9me3 peptide. Kd is shown below. (**c**) *In vitro* pulldown assay showing that Chp1CD interacts with the core of the nucleosome and that LOOP1B/2B mutations abolish this interaction. (**d**) *In vitro* pulldown assay showing Chp1CD interaction with H3K9me3Nucleosomes. LOOP1B/2B mutations strongly reduce the interaction with H3K9me3Nucleosomes. (**e**) Thermophoresis assay showing binding curves of wild-type Chp1CD and LOOP1B/2B Chp1CD mutant to H3K9me3Nucleosomes. Kd is shown below the image. (**f**) *In vitro* pulldown assay showing that Chp1CD-H3K9me3Nucleosome complex interacts with RNA. Chp1CD LOOP1B/2B mutations abolished this interaction.

We used our structural data to design mutations in two loops of the Chp1CD that interact with the core of the nucleosome. In the Chp1CD LOOP1B construct we mutated two residues of the primary interacting loop (N33A and N35A) that are in proximity of the H3 loop. LOOP1A construct has R31S mutation in addition to N33A and N35A. The Chp1CD LOOP2B construct has W49A, Y50A and D51A mutated, whereas the Chp1CD LOOP2A has a N52A point mutation (Figure 4a). In our model, these residues are in the proximity of the nucleosome and likely interact with the core. Sequence alignments indicate that residues R31, D51 and N52 are conserved in other chromodomains in *S. pombe* and other eukaryotes, whereas N33 and N35 are conserved only in a subset of chromodomains (Supplementary Table S1A). The residues of LOOP1B that were mutated are also conserved in the Sir3BAH domain from *Saccharomyces cerevisiae* (Supplementary Table S1B) and interact with the nucleosome [30–33].

We purified the above described Chp1CD mutants (Supplementary Figure S5A) and confirmed that the interaction with the H3K9me3 peptide is unaffected for Chp1CD LOOP1B, 2A and 2B constructs. We observed partial defect in binding to H3K9me peptide in LOOP1A construct (R31S mutation), whereas the previously characterized mutant W44A shows a complete loss of interaction (Supplementary Figure S5B and C). As LOOP1A mutant showed reduced binding to H3K9me peptide, we have focused on LOOP1B and 2B mutants for further analysis. We confirmed our initial peptide pull-down experiments by thermophoresis, an equilibrium binding assay [34]. Thermophoresis showed no change in binding to the H3K9me peptide for LOOP1B and 2B mutants (Figure 4b; Supplementary Figure S5D). The peptide-binding assays also show that Chp1CD LOOP1B and 2B mutants are properly folded and capable to specifically interact with H3K9me tail (Figure 4b; Supplementary Figure S5). These mutants also show no defect in the interaction with RNA (Supplementary Figure S6A) [17].

We generated nucleosomes without histone tails (tailless) to determine whether Chp1CD mutations specifically abolish interaction with the core of the nucleosome (Supplementary Figure S6B and C). *In vitro* binding assays reveal that wild-type Chp1CD can weakly bind to the nucleosome core even in the absence of histone tails (Figure 4c). Chp1CD LOOP1B/ 2B mutations resulted in a non-detectable interaction with the nucleosome core (Figure 4c; Supplementary Figure S6D). These data show that the two loops of Chp1CD form the main binding interface with the nucleosome core, and mutation in LOOP1B and 2B specifically abolish the interaction. A similar pattern was observed with unmodified nucleosomes (Supplementary Figure S6F).

As expected, wild-type Chp1CD had the highest affinity for H3K9me3Nucleosomes in our binding assays. When LOOP1B and 2B were mutated, we observed a strong reduction in binding to H3K9me3Nucleosomes by pulldown assay (Figure 4d; Supplementary Figure S6E). Although the Chp1CD LOOP1B/2B mutant has similar affinity for the H3K9me tail as wild-type Chp1CD (Figure 4b; Supplementary Figure S5D), the binding to the H3K9me3Nucleosomes was clearly reduced. These data show that binding to the nucleosome core further stabilizes the Chp1CD-H3K9me3Nucleosome complex (Figure 4d). We have confirmed our finding by an equilibrium thermophoresis assay and observed more than fivefold reduction in Kd to H3K9me3Nucleosomes when LOOP1B and 2B were mutated (Figure 4e). Mutating H3K79A/T80A/D81A on the nucleosome surface was not sufficient to abolish the interaction with the H3K9me3Nucleosomes, likely because of several interaction points between Chp1CD and the nucleosome core (Supplementary Figure S6G). This is similar to the observation that Sir3 binding to nucleosome was not affected by H3K79A mutation, but only by H3K79me [31].

Although Chp1CD LOOP1B/2B had no defect in binding to RNA (Supplementary Figure S6A), RNA binding to Chp1CD_LOOP1B/2B-H3K9me3Nucleosome complex was severely impaired (Figure 4f). Our data show that Chp1CD interaction with the core is also important to stabilize nascent transcripts tethering to chromatin.

Both, the cryo-EM structure and *in vitro* binding assays indicate that Chp1CD, after recognition of H3K9me tail, interacts with the core of the nucleosome and this interaction increases the stability of the complex and promotes interaction with RNA.

### Chp1CD binding to the core is required for heterochromatin formation

**Figure 5:**
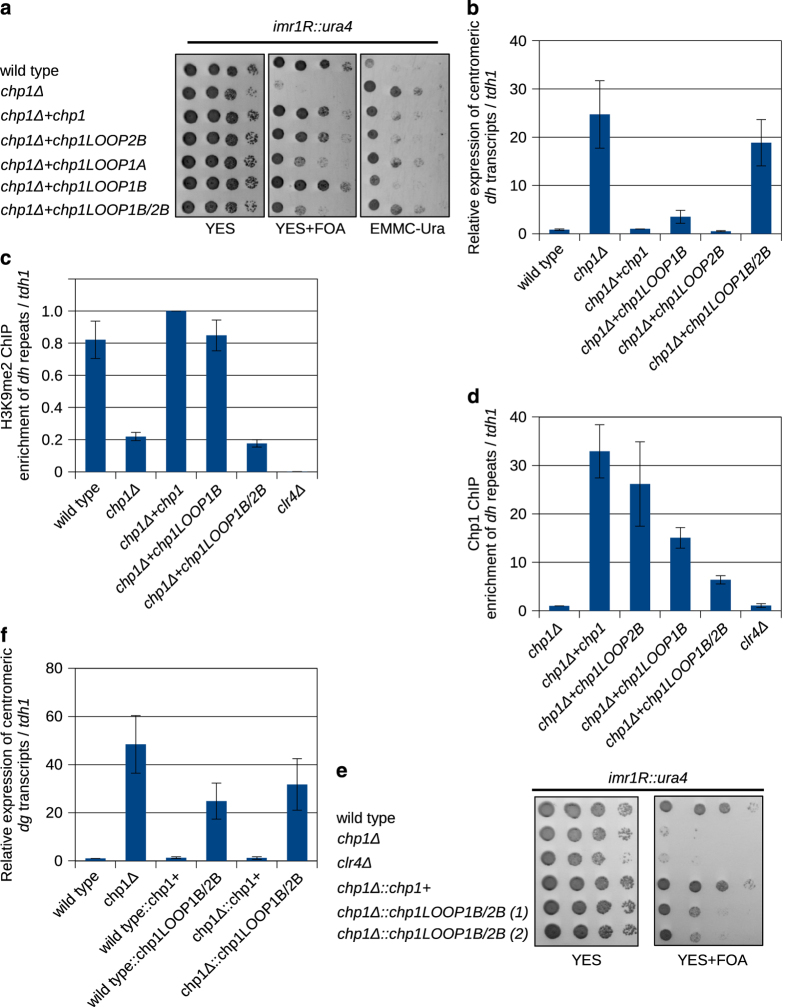
Chp1CD interaction with the core of the nucleosome is required for heterochromatin formation. (**a**) Silencing assay showing that *Chp1CD LOOP1B/2B* mutant cells have a defect in heterochromatin formation at centromeric repeats. Tenfold serial dilutions were spotted on YES, YES+FOA and EMMC-Ura plates. (**b**) Relative expression of centromeric *dh* transcripts in wt and *Chp1CD* mutant cells. Yeast cells with *Chp1CD LOOP1B/2B* mutations show accumulation of pericentromeric *dh* transcripts to the levels of *chp1Δ*. Error bars indicate s.e.m. (**c**) ChIP experiment showing that H3K9me is reduced at centromeric *dh* repeats in *Chp1CD LOOP1B/2B* mutant cells to the level of *chp1Δ*. Error bars indicate s.e.m. (**d**) ChIP experiment showing that Chp1CD LOOP1B and 2B mutants are less efficiently recruited to centromeric *dh* repeats. Error bars indicate s.e.m. (**e**) Silencing assay showing that genomically integrated *Chp1CD LOOP1B/2B* mutant cells have a defect in heterochromatin formation at centromeric repeats. Tenfold serial dilutions were spotted on YES and YES+FOA plates. (**f**) Relative expression of centromeric *dg* transcripts in wt and genomically integrated *Chp1CD* mutant cells. Yeast cells with Chp1CD LOOP1B/2B mutations show accumulation of pericentromeric *dg* transcripts to the levels of *chp1Δ*. Error bars indicate s.e.m.

To determine whether the interaction with the nucleosome core is required for Chp1 chromodomain function in heterochromatin formation, we have reintroduced wild-type and mutant *chp1* genes into *chp1Δ* fission yeast cells. All *chp1* constructs were expressed at similar level (Supplementary Figure S7A). Reintroduction of wild-type *chp1* into *chp1Δ* cells re-established fully functional pericentromeric heterochromatin (Figure 5a; Supplementary Figure S7B). To the contrary, reintroduction of *chp1LOOP1B/2B* mutants into *chp1Δ* cells did not result in pericentromeric heterochromatin formation (Figure 5a). Consistent with the growth assays, *chp1LOOP1B/2B* mutant cells show complete loss of silencing at pericentromeric *dh* repeats and almost complete de-repression of pericentromeric *dg* transcripts (Figure 5b; Supplementary Figure S7B–D). We also observed a reduction in H3K9me at centromeric repeats in *chp1LOOP1B/2B* mutant to the level of *chp1Δ* cells (Figure 5c; Supplementary Figure S8A). Wild-type Chp1 binds centromeric repeats as shown by chromatin immunoprecipitation. When we mutated Chp1 chromodomain loops 1B and 2B, we observed strong reduction in recruitment to centromeric heterochromatin (Figure 5d; Supplementary Figure S8B). To confirm our findings with the plasmid rescue, we integrated *Chp1* and *Chp1LOOP1B/2B* into the native *chp1+* locus in wild-type and *chp1Δ* cells. Genomic integration of *LOOP1B/2B* shows a similar loss of centromeric silencing like the one observed in the plasmid rescue assay (Figure 5e). Consistent with the growth assays, genomic integration of *LOOP1B/2B* mutant shows almost complete de-repression of pericentromeric *dg* and *dh* transcripts (Figure 5f; Supplementary Figure S8C). Similar result was obtained when we integrated *Chp1LOOP1B/2B* mutant into wild-type cells or into *chp1Δ* cells. Integration of *Chp1* and *Chp1LOOP1B/2B* mutant into wild-type cells shows that pure maintenance of heterochromatin is defective (Figure 5f; Supplementary Figure S8C). Integration of *Chp1* and *Chp1LOOP1B/2B* into *chp1Δ* cells shows that *Chp1CD LOOP1B/2B* mutant is also defective in re-establishment of heterochromatic silencing. To test *de novo* establishment of centromeric heterochromatin, we generated *clr4Δ* and *clr4Δchp1LOOP1B/2B* mutants cells and subsequently reintroduced *clr4* gene [35]. After reintroduction of *clr4* gene, *clr4Δchp1LOOP1B/2B* cells did not re-establish centromeric silencing, indicating that binding to the nucleosome core is required for heterochromatin establishment as well (Supplementary Figure S8D).

Our data indicate that Chp1 interaction with the nucleosome core is essential for stable binding of Chp1 to H3K9-methylated nucleosomes and for heterochromatic silencing. We show that LOOP1B/2B mutant has no defect in binding to H3K9me tail, but shows reduced binding to H3K9me3Nucleosomes *in vitro*. Although expressed at similar level as the wild-type protein, the mutant shows reduced localization to *dh* and *dg* repeats *in vivo* and loss of silencing at centromeric repeats. Our data show that Chp1CD binding to the nucleosome core is required for heterochromatin formation.

## Discussion

The organization of chromatin into heterochromatin is important for a wide range of cellular processes such as epigenetic gene regulation, nuclear organization and chromosome segregation. H3K9me is recognized by the chromodomain, a conserved protein module essential for targeting of diverse proteins to chromatin and for heterochromatin formation. When the HP1 chromodomain was replaced with the polycomb chromodomain, HP1 was mis-localized to polycomb binding sites in *Drosophila* [5]. Fission yeast cells having Clr4CD replaced by mutant Chp1CD were not able to spread the heterochromatin beyond initiation sites [22]. Also, swapping Swi6CD with Chp1CD or Chp2CD impaired centromeric heterochromatin and silencing, although all three chromodomains recognize H3K9 methylation [17, 21]. These data show that the chromodomain interaction with H3K9me3Nucleosomes, and not only with the peptide, determines how the distinct functions of diverse chromodomain-containing proteins are coordinated at the same locus. The structural features that allow recognition of H3K9-methylated Nucleosomes by chromodomains are not well understood and are central to understand the molecular mechanisms of heterochromatin assembly.

To address these questions, we have solved the cryo-EM structure of the fission yeast Chp1CD bound to H3K9me3Nucleosomes. The structure shows that chromodomains interact with both the H3K9me tail and the core of the nucleosome. The main interaction with the nucleosome core is the histone H3 region 77–81aa and acidic patch formed by H2A and H2B. Histone 3 lysine 79 (H3K79) is methylated in *S. cerevisiae* and mammalian cells by Dot1 methyltransferase [13, 36, 37]. It has been shown that H3K79 methylation antagonizes binding of the silencing protein Sir3 to the nucleosome in *S. cerevisiae* [31, 32, 38, 39]. Dot1 homolog is not present in *S. pombe* and it remains open whether posttranslational modifications of H3K79 could regulate the Chp1CD interaction with the nucleosome. Several additional residues (H3T80, H2BK108, H4K55, H4K59) in regions of the nucleosome core that are interacting with Chp1CD are posttraslationaly modified in human cells [40, 41]. The posttranslational modifications in the nucleosome core could provide additional specificity in Chp1 and RITS recruitment to the chromatin and another layer of regulation in initiation of heterochromatin formation. Our data imply that the chromodomain interaction with the nucleosome could also be regulated by a histone code in the nucleosome core. This suggests that a single chromodomain could read multiple histone marks.

Establishment of centromeric heterochromatin in fission yeast is mediated by RNA interference. Our data show that Chp1CD binding to the nucleosome core has an essential role in heterochromatin establishment. In the establishment step, small RNAs guide Argonaute to nascent centromeric transcripts to deposit first H3K9me mark [35, 42]. This will increase the concentration of Chp1 at the centromeric locus and recruit Chp1CD to the nucleosome core. The recruitment to the core will stabilize interaction between RITS and chromatin at the initial steps of heterochromatin establishment in an H3K9me-independent manner. This will deposit first H3K9me mark and tether RITS complex to chromatin to initiate generation of high siRNAs levels. This is consistent with previous study showing that reduction in Chp1CD affinity for H3K9me also impairs establishment of heterochromatin [10]. Our data show that high affinity of Chp1CD for the nucleosome is provided by binding both the H3K9me tail and the nucleosome core.

**Figure 6:**
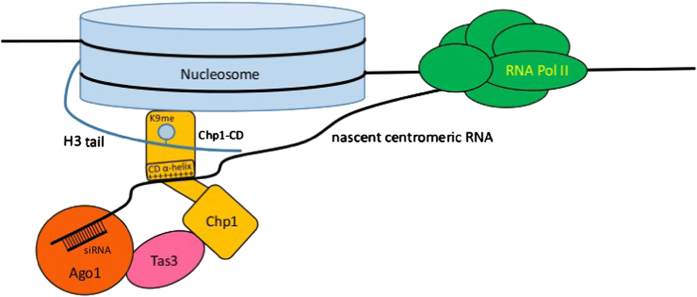
Chp1CD interaction with the core of the nucleosome is required for heterochromatin formation. Model showing Chp1CD interaction with the H3K9me3Nucleosome. In establishment mode, Chp1CD will be tethered to centromeric repeats by sRNAs and Argonaute. Chp1CD will bind to the core of the nucleosome. This will tether Argonaute to chromatin even in absence of H3K9me. After deposition of initial H3K9me, Chp1 will bind H3K9me histone tail to further stabilize interaction with the chromatin. The interaction with the nucleosome core is required for heterochromatin formation and silencing of centromeric repeats in fission yeast.

In the maintenance mode, existing heterochromatin has to be propagated through cell divisions. Although high affinity of Chp1CD for H3K9me peptide is not essential for heterochromatin maintenance [10], we observe almost complete loss of heterochromatin maintenance at centromeric repeats in our mutants. This suggests that Chp1CD binding to the core might be required for downstream processes in RNA interference-mediated heterochromatin maintenance. It has been shown that Chp1CD binds RNA and DNA *in vitro* with the same affinity. After binding to the H3K9me peptide, Chp1CD affinity for RNA and DNA was increased, suggesting that Chp1CD will bind nascent RNA transcripts or DNA after association with the H3K9-methylated Nucleosome [17]. An interaction with the DNA in the context of the nucleosome core particle was not observed in the cryo-EM structure. In the structure, Chp1CD binds the nucleosome solely through protein–protein interactions with histones and the positively charged α-helix, suggested to bind RNA, faces outwards. Our *in vitro* data show that Chp1CD can indeed bind RNA when bound to the H3K9me3Nucleosomes. To the contrary, Chp1CD_LOOP1B/2B-H3K9me3Nucleosome complex shows strong reduction in affinity for RNA. This indicates that interaction with the nucleosome core prevents unspecific binding of positively charged α-helix to DNA and promotes interaction with RNA (Figure 6). Consistent with our findings, mutations in the positively charged α-helix had a defect in heterochromatin formation, indicating that RNA tethering to chromatin is required for efficient silencing in fission yeast [17].

Our structure and biochemical assays show that Chp1CD binding to H3K9me tail tethers it to to the core of the nucleosome. The interaction with the core stabilizes the Chp1CD-H3K9me3Nucleosome complex and primes it for interaction with RNA. We show that binding to the core is required for Chp1 recruitment to the chromatin and heterochromatin formation at centromeric repeats in fission yeast. Our data show that the chromodomain interaction with the nucleosome is far more complex than just an interaction with the histone tail. The interaction with the nucleosome core can also explain why the swapping of one chromodomain with another chromodomain recognizing the same H3K9 methylation mark leads to the loss-of-function phenotype [17, 21, 22]. In addition, the chromodomain interaction with the nucleosome core could provide another layer of regulation for the association with the chromatin by recognizing histone marks in the core. This might explain how different functions of the various chromodomain-containing proteins are coordinated at the same locus.

## Materials and Methods

### Plasmids and strains construction

Chp1CD mutants for expression and purification were generated through inverse PCR using the primers listed in Supplementary Table S2 starting from the pET28a Chp1 chromodomain plasmid [10]. Heterochromatin rescue assays were performed by introduction of plasmids containing *chp1* wt and *chp1* mutant protein under its endogenous promoter in a *chp1Δ* strain (SP170, see Supplementary Table S3) [35]. *chp1* full-length gene and its endogenous promoter (−949 bp from the start of *chp1+* coding sequence) were cloned in the pREP1 plasmid. *chp1* chromodomain mutants were generated by inverse PCR using the primers listed in Supplementary Table S2 and transformed in the indicated *chp1Δ* strain.

For genomic integration, the pREP1 plasmid was modified to replace the *nmt1+* promoter with the following integration cassette: (SphI) Region at the 5′ of Chp1 gene (Chromosome I, 2215500–2215055) (AscI)—*HphMX6* resistance cassette -(SphI) Chp1 endogenous promoter (Chromosome I, 2214829–2214664)—Chp1 coding sequence—(BamHI) Chp1 terminator (Chromosome I, 2210976–2210582) (BamHI). The integration cassette (both the *chp1+* and the *chp1LOOP1B/2B* cassette) was then PCR amplified and transformed by electroporation, into SP101, SP170 and SP64 strains. Cells were then selected on YES+ Hygromycin (50 mg ml^−1^ Hygromycin) plates. Single colonies were isolated, PCR screened and sequenced for the genomic insertion of the *HphMX6* resistance cassette and the LOOP1B/2B mutations.

Strains containing plasmids were grown on Edinburgh Minimal Medium Complete (EMMC)-leu media. All strains and plasmids used in this study are listed in Supplementary Tables S3 and S4.

### Protein purification

His_6_-SUMO-Chp1CD and all CD mutants were expressed in *E. coli* BL21 (DE3)(pLys) and purified through affinity chromatography by using Ni-NTA resin (GE Healthcare, Freiburg, Germany). His_6_-SUMO-Chp1CD contains thrombin cleavage site between two tags [10].

In total, 0.2 mM IPTG was added to induce protein expression followed by growth at 18 °C O/N. Cells were harvested by centrifugation and re-suspended in lysis buffer (20 mM 4-(2-hydroxyethyl)-1-piperazineethanesulfonic acid (HEPES) pH 7.5, 150 mM NaCl, 1 mM dithiothreitol (DTT), 20 mM imidazole). After flash freezing, cells were thawed and incubated for 30 min in lysozyme before sonication (Branson Sonifier 250 -output 4, duty cycle 40). The suspension was centrifuged (12000 g, 20 min at 4 °C) and the supernatant added to the Ni-NTA resin pre-equilibrated in binding buffer (20 mM HEPES pH 7.5, 500 mM NaCl, 1 mM DTT, 20 mM imidazole) and incubated for 30 min at 4 °C under rotation. Resin was then 5× washed with binding buffer and proteins were eluted in elution buffer (20 mM HEPES pH 7.5, 150 mM NaCl, 1 mM DTT, 300 mM imidazole). Chp1CD wt and mutants were then dyalized O/N in a buffer containing 20 mM HEPES pH 7.5, 150 mM NaCl, 1 mM DTT. His_6_-SUMO-Chp1CD was then further purified by gel filtration (Superdex 75 pg; GE Healthcare) and dialyzed in a buffer containing 20 mM HEPES pH 7.5, 75 mM KCl, 0.5 mM DTT.

### Silencing assays

Cells for silencing assays were grown to a OD 0.7–1 and then normalized to a final concentration of 1×10^7^ cells ml^−1^ of culture. Tenfold serial dilutions were made so that the highest density spot contained 1×10^5^ cells. Cells were spotted on non-selective (YES) and 5-fluoro-orotic acid (5-FOA 1 g l^−1^ 5-FOA) plates. The plates were incubated at 32 °C for 2–3 days and imaged. Cells have a ura4 reporter gene inserted into pericentromeric *imr* repeats (heterochromatic locus). When *ura4* reporter gene is silenced, cells can grow on 5-FOA containing medium. When heterochromatin is lost, *ura4* reporter gene is expressed and cells are unable to grow on 5-FOA medium.

### Detection of RNA levels by qPCR (RT-qPCR)

Yeast cultures (10 ml) were grown to an OD600 of 0.7–1.5. Cells were then re-suspended in 500 μl lysis buffer (300 mM NaOAc pH 5.2, 1% sodium dodecyl sulfate) and 500 μl phenol–chloroform and incubated at 65 °C for 10 min with constant mixing. The aqueous fraction was separated from phenol–chloroform by centrifugation (10 min, 20 000 g) and ethanol precipitated. Nucleic acids were treated with DNAse I (Roche, Basel, Switzerland) for 30 min at 37 °C followed by 15 min at 75 °C heat inactivation. Complementary DNA was synthesized using 100 ng of RNA and 1 pmol of DNA oligos with Superscript III (Invitrogen, Darmstadt, Germany) using standard conditions. RNA levels were quantified with qPCR by using the Biozym DyNAmo Flash qRT-PCR kit and normalized to euchromatic gene *tdh1*.

### RNA electrophoretic mobility shift assays

RNA shift assay were performed following the conditions previously reported [17]. In total, 0.66 pmols of ^32^P radiolabeled 30 nt centromeric RNA were incubated with 10 μM Chp1 chromodomain (wild-type and mutant) in a buffer containing 20 mM Tris-HCl pH 7.5, 100 mM KCl, 0.5 mM DTT and 3% Glycerol. For the RNA EMSA with 100 nt *dg* centromeric transcripts, 2 pmols of ^32^P radiolabeled RNA were incubated with 0, 2 (1:1), 10 (1:5), 20 (1:10) pmols of Chp1CD protein (wt and LOOP1B/2B mutant) in 15 μl final volume. H3K9me3 peptide (Eurogentec, Köln, Germany) was added at a 1:1 Chp1CD- H3K9me peptide, as reported in [17]. Incubation was carried out for 1 h on ice and samples (20 μl final volume) were then loaded on a 10% Acrylamide-TBE Native gel (Bis-Acrylamide ratio 1:29). For the *in vitro* RNA pull-downs, 1 μg of SUMO-Chp1CD (wild-type and LOOP1B/2B mutant) was bound to 15 μl Ni-NTA resin (GE Healthcare) and the H3K9me3Nucleosome was added to assemble the Chp1CD-H3K9me3Nucleosome complex in Binding buffer (20 mM HEPES pH 7.5, 75 mM KCl, 0.5 mM DTT, 20 mM imidazole). After washing three times with 50 μl of binding buffer, 2 pmols of 32P-labeled 100nt *dg* RNA were added and incubated with the Chp1CD-Nucleosome resin on ice for 1 h. The resin was centrifuged at slow speed (60 g, 10 s) and the flow-through collected. Resin was washed for three times with at least 3× (v/v) Binding buffer and the Chp1CD-Nucleosome-RNA complex was eluted by addition of 300 mM imidazole buffer (20 mM HEPES pH 7.5, 75 mM KCl, 0.5 mM DTT, 300 mM imidazole), incubated for 1 h on ice with 5 min interval mixing (bound fraction). Samples were loaded on a 10% TBE Native Acrylamide gel (Bis-Acrylamide ratio 1:29) and run for 2 h at 10 mA at 4 °C. After overnight exposition, gels were scanned using the TyphoonFLA9000 phosphoimager.

### Chromatin immunoprecipitation

Yeast cultures (100 ml) were grown to an OD600 of 0.7 and cross-linked with 3% formaldehyde at room temperature for 15 min as described [35]. The reaction was quenched with 125 mM glycine for 10 min at room temperature. Cells were re-suspended in 500 μl lysis buffer (50 mM HEPES pH 7.5, 1.5 M sodium acetate, 5 mM MgCl_2_, 2 mM ethylenediaminetetraacetic acid, 2 mM ethylene glycol tetraacetic acid, 0.1% NP-40, 20% glycerol) containing protease inhibitors (protease inhibitor cocktail tablets, Roche, Complete, ethylenediaminetetraacetic acid free). Frozen cells were lysed using the MP Biospec bead beater. After lysis, the extract was sonicated 35× for 30 s (Bioruptor, Diagenode, Seraing, Belgium) and spun at 13 000 g for 15 min to obtain the chromatin supernatant. For input DNA, 50 μl of the supernatant were used. For immunoprecipitations, the supernatants were normalized based on the protein concentration and incubated with anti-dimethylated H3K9 antibody or anti-Chp1 antibody (H3K9me2, Abcam no. Ab1220 and Chp1, Abcam no. Ab18191), immobilized on magnetic Dynabeads, for 2 h at 4 °C. The beads and immobilized protein were washed 5× with 1 ml of lysis buffer. Proteins were eluted by incubating with 150 μl of elution buffer (50 mM Tris-HCl pH 8.0, 10 mM ethylenediaminetetraacetic acid, 1% sodium dodecyl sulfate) at 65 °C for 15 min. Cross-links were reversed by incubation at 65 °C overnight followed by RNA degradation with RNase A and protein degradation with Proteinase K. DNA was then recovered by phenol–chloroform extraction and ethanol precipitation and quantified using qPCR. Euchromatic gene *tdh1* was used for normalization. Oligonucleotides used in chromatin immunoprecipitation assays are listed in Supplementary Table S2.

### Nucleosome *in vitro* reconstitution and (H3K9me3) methylation

Nucleosomes were reconstituted using *Xenopus laevis* histones and the 601 sequence [29, 43] as previously described [26, 27]. *In vitro* methylation was done as described [27]. The peak at +42 Da has been observed in original publication and it is not a contamination (Matt Simon, personal communication and described in [27]).

### *In vitro* Chp1CD–H3K9me3 MLA Nucleosome complex formation and elution

In total, 5 μg of Chp1CD were bound to 15 μl of Ni-NTA resin in binding buffer (20 mM HEPES pH 7.5, 75 mM KCl, 0.5 mM DTT, 20 mM imidazole) for 20 min at 4 °C. The resin was washed once with five volumes of binding buffer and 10 μg of H3K9me3 methyl lysine analog (MLA) nucleosomes were added (MLA nucleosomes were previously dialyzed in binding buffer) in a final volume of 20 μl and incubated 1 h on ice with constant re-suspension every 5 min. After incubation, the resin was centrifuged (60 g for 10 s) and the flow-through collected. The resin was then washed three times with five volumes of binding buffer. The SUMO-Chp1CD-H3K9me3Nucleosome complex was eluted by adding thrombin (Sigma, Munich, Germany) for 2 h on ice in 20 μl of binding buffer. Complex formation was then assessed through SDS-polyacrylamide gel electrophoresis on 15% acrylamide gels and by negative stain EM.

### Chp1CD H3K9me3 peptide-binding assays

In total, 1 μg of [Lys(Me3)9]-Histone H3 (1–21)-GGK(Biotin) peptide (Eurogentec) was incubated with 15 μl of streptavidin agarose resin (Invitrogen) in 20 mM HEPES pH 7.5, 75 mM KCl, 0.5 mM DTT. Approximately 5 μg of Chp1CD (both wt and mutants) were then added in a final volume of 20 μl and incubated for 1 h on ice under the same buffer conditions. The resin was washed three times and then the binding efficiency was assessed on SDS-polyacrylamide gel electrophoresis on 15% acrylamide gels. Quantification of binding was done by using the ImageJ software. Binding of each mutant was normalized to wt Chp1CD for each assay.

### Microscale thermophoresis (MST)

For MST SUMO tag was removed from Chp1CD constructs with Ulp1 protease. In total, 100 μg of wild-type and mutant Chp1CD were fluorescently labeled using the MO-L003 Monolith Protein Labeling Kit BLUE-NHS (Amine Reactive) according to manufacturer's instructions (Nanotemper Technologies, Munich, Germany). A 1:1 Fluorescence: Protein ratio was estimated by using the Nanodrop1000 software ‘Proteins and Labels’ feature and each Chp1 Chromodomain was run on a 15% SDS acrylamide gel to normalize concentrations to 0.1 mg ml^−1^.

Reactions were assembled in 20 μl with 300 ng of fluorescent Chp1CD, and increasing quantities of [Lys(Me3)9]-Histone H3 (1–21)-GGK(Biotin) peptide (Eurogentec) or H3K9me3Nucleosomes, in a buffer containing 10 mM Tris-HCl pH 7.5, 150 mM NaCl, 0.5 mM DTT and 0.05% Tween-20. For H3K9me3Nucleosomes-binding assays glycerol was added to 10% final concentraction. For H3K9me3 assays these dilutions were used for measurements: 0 nM, 9 nM, 13 nM, 20 nM, 31 nM, 46 nM, 70 nM, 105 nM, 158 nM, 237 nM, 355 nM, 530 μM, 800 μM, 1.2 μM, 1.8 μM. For H3K9me3Nucleosome assay we used following dilutions for measurements: 0 nM, 18 nM, 27 nM, 41 nM, 62 nM, 93 nM, 140 nM, 210 nM, 316 nM, 474 nM, 711 nM, 1.06 μM, 1.2 μM, 1.4 μM, 1.6 μM, 2.4 μM.

MST runs were performed using standard treated capillaries (Nanotemper Cat#K002) on the NT.115 Monolith instrument. All measurements were performed using 80% LED and 40% MST power, with 30 s Laser On time and 5 s Laser Off time. For each experiment, five single measurements were performed.

Data were analyzed with the GraphPad Prism software version 6.00 (GraphPad Software, San Diego, CA, USA) and the SigmaPlot software version 13.0 (Systat Software, San Jose, CA, USA). For the peptide-binding assays, with the curves showing a distinct sigmoid trend, we fitted the Richard´s five Parameter Logistic Asymmetric Sigmoidal equation and automatically calculated the Kd. For the H3K9me3Nucleosome-binding assays, as the raw data trend was not anymore sigmoid for all proteins analyzed, a third order polynomial (cubic) equation was used for fitting and comparing the raw data points of wild-type Chp1CD and the different mutants. Kd was calculated based on the ‘Interpolation’ feature of the GraphPad prism software, using the 50% bound/unbound value on the *y* axis.

### Nucleosome trypsin digestion

Tailless nucleosomes were prepared by incubating reconstituted nucleosomes with an immobilized TPCK-Trypsin resin (Thermoscientific) for 2 h at room temperature in a buffer containing 20 mM HEPES pH 7.5, 75 mM KCl, 0.5 mM DTT [44]. The tryptic digestion generates very defined histone bands and it has been characterized in detail where exactly trypsin cuts [45].

### Nucleosome-binding assays with Chp1CD mutants

Chp1CD wild-type and mutant nucleosome-binding assays were performed as described earlier for the Chp1CD—H3KC9me3 MLA Nucleosome complex formation in 20 mM HEPES pH 7.5, 100 mM KCl, 0.5 mM DTT, 40 mM imidazole. Resins and INPUTs were run on SDS-polyacrylamide gel electrophoresis 15% acrylamide gels. All samples were then analyzed by immunoblot with anti-H3 histone (AbCam, Cambridge, UK, 1:1000), or anti-H3K9me3 Antibody (AbCam, 1:1000), anti-goat IgG-HRP (BioRad, 1:3000) anti-rabbit IgG-HRP (BioRad, Munich, Germany, 1:3000).

### Negative stain electron microscopy

After thrombin elution, 3 μl of the Chp1CD–H3KC9me3 nucleosomes complex were spotted on a glow-discharged copper grid (Cu 400 mesh Q11916, Quantifoil, Großlöbichau, Germany) coated with a 1 nm carbon film for 45 s. After a quick wash with water, the grid was incubated for 15 s in 2% Uranyl Acetate. Negative stain images were collected on a FEI Morgagni transmission electron microscope.

### Cryo-EM on the Chp1CD–H3KC9me3 nucleosomes complex

Cryo-EM grids (Holey carbon-coated grids, Cu 300 Mesh R3/3+1 nm carbon layer, Quantifoil) were prepared using a Vitrobot Mark IV (FEI Company). Cryo-EM data were collected using a Titan-Krios transmission electron microscope (FEI Company, Hillsboro, OR, USA) at 200 KeV and a magnification of 113 000× at the plane of the CCD using a F816 CMOS camera (TVIPS GmbH, Gauting, Germany) resulting in an image pixel size of 1.2 Å per pixel on the object scale (pixel size of the CCD was 13.6 μm). For automated data collection the EM-TOOLS software was used and data were collected in a defocus range of 10 000–40 000 Å.

In total, 2 480 micrographs for the Chp1CD–H3KC9me3 complex and 991 micrographs for the H3KC9me3 nucleosome control, respectively, were selected for single-particle analysis using the Xmipp software package (Supplementary Figure S9A) [46]. Few thousand particles were manually picked and carefully cleaned from noise. These particles were then used for semi-automatic and automatic particle picking in XMIPP. Contrast transfer function was determined by CTFFIND3 [47]. Selected single particles were converted to SPIDER and RELION formats for further analysis (Supplementary Figure S9B). The two-dimensional class averages were generated with RELION software package (Supplementary Figure S9C) [48]. Bad class averages were removed from further data analysis. The three-dimensional refinements were subsequently done with the SPIDER and RELION software packages [49].

Unsupervised particle classification was performed by random seeding with the complete density maps without focused classification in SPIDER software package. Attempts to use focused classification resulted in a strong bias and noise overfitting in regions of interest. Therefore, we did not to use focused classification to reduce bias. In initial classification done in SPIDER, classes C0–C6 and N0–N5 were backprojected using angles from C0 and N0 maps and this classes were not fully refined. Here we just wanted to select classes that had additional density bound to the nucleosome. Particles generating maps with different Chp1CD densities were separated and further classified. Approximately 20 rounds of random seeding classifications were performed until we have partitioned particles into five different groups (Supplementary Figure S3). Classes C11–C15 were refined with RELION software package. Final refinements of Chp1CD-H3K9me3Nucleosome complexes (C15 class) and Nucleosome control were done with RELION software package. For final refinement reference was filtered to ~50 Å (RELION filter). The reference we used had none of the features observed in refined reconstructions (the DNA double helix is not resolved, major groove is not visible, α-helices are not resolved). This indicates no reference bias in our structure. The resolution of Nucleosome control reached 7.3 Å using auto-refine in RELION and C2 symmetry (FSC 0.143 cutoff of two independently refined maps). Chp1CD-H3K9me3Nucleosome complex was refined to 10 Å (FSC 0.143 cutoff of two independently refined maps) with no symmetry applied.

Local resolution was calculated using ResMap software [28] (final single volume, minRes=7, maxRes=14, automask). Mean resolution determined by Resmap is 9.4 Å for Chp1CD-H3K9me3Nucleosome complex, independently confirming previously determined average resolution of 10 Å (FSC 0.143) (Figure 1c). For Chp1CD-H3K9me3Nucleosome complex local resolution for nucleosome is 9–10 Å and for the ligand ~10 Å (Figure 2a). Euler angle distribution for final reconstructions is shown for both Nucleosome and Chp1CD-H3K9me3Nucleosome complex (Supplementary Figure S9D). All orientations are present with a preference for top and side views.

Molecular models were built using Chimera software package using rigid body fitting of crystal structures [50]. The fit into density was done with Chimera options ‘Fit in Map’ and ‘Fit in Segments’ with only minor manual adjustments. Segmentation and visualization of all cryo-EM maps was done with Chimera software as well [50].
