# Supplementary material for: The Chp1 chromodomain binds the H3K9me tail and the nucleosome core to assemble heterochromatin
Source: Cell Discov. 2016 Apr 19;2:16004–. doi: 10.1038/celldisc.2016.4 (PMC4849473; doi:10.1038/celldisc.2016.4)
Supplement: Supplementary Figure S1 [file celldisc20164-s1.pdf]

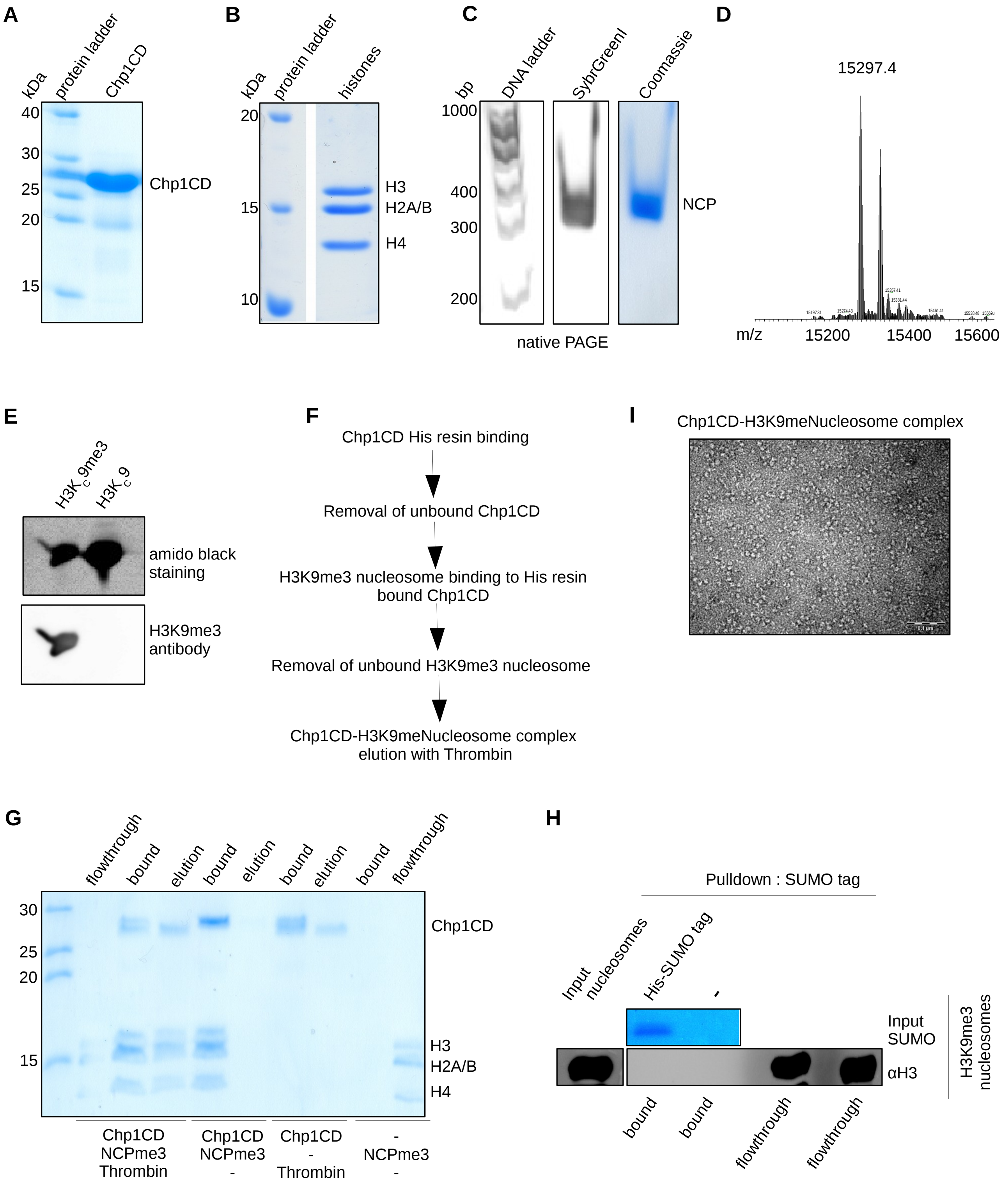
A
kDa
protein ladder
Chp1CD
40
30
25
20
15
Chp1CD
B
kDa
protein ladder
histones
20
15
10
H3
H2A/B
H4
C
bp
DNA ladder
SybrGreenI
Coomassie
1000
400
300
200
NCP
native PAGE
D
15297.4
m/z
15200
15400
15600
E
H3K$_C$9me3
H3K$_C$9
amido black staining
H3K9me3 antibody
F
Chp1CD His resin binding
Removal of unbound Chp1CD
H3K9me3 nucleosome binding to His resin bound Chp1CD
Removal of unbound H3K9me3 nucleosome
Chp1CD-H3K9meNucleosome complex elution with Thrombin
I
Chp1CD-H3K9meNucleosome complex
G
flowthrough
bound
elution
bound
elution
bound
elution
bound
flowthrough
30
25
20
15
Chp1CD
H3
H2A/B
H4
Chp1CD NCPme3 Thrombin
Chp1CD NCPme3 -
Chp1CD - Thrombin
- NCPme3 -
H
Pulldown : SUMO tag
Input nucleosomes
His-SUMO tag
-
Input SUMO
αH3
H3K9me3 nucleosomes
bound
bound
flowthrough
flowthrough

Figure S1

Figure S1. Reconstitution of the Chp1CD-H3K9me3Nucleosome complex (H3K9me3NCP).

(A) Coomassie stained SDS polyacrylamide gel showing purified chromodomain of Chp1.

(B) Coomassie stained SDS polyacrylamide gel showing purified *Xenopus leavis* histones H2A, H2B, H3 and H4.

(C) Coomassie and SybrGreen II stained native polyacrylamide gel showing reconstituted nucleosomes.

(D) Mass Spectrometry (MS) analysis of $H3K_C9me3$ histone showing a peak at 15297 Da. The MS analysis is consistent with previously published results.

(E) Western blot showing that $H3K_C9me3$ histone is specifically recognized by the H3K9me3 antibody.

(F) Scheme showing the strategy we used to assemble the Chp1CD-H3K9me3Nucleosome complex.

(G) Assembly of the Chp1CD-H3K9me3Nucleosome complex (NCPme3). Coomassie stained SDS polyacrylamide gel showing the complex elution from the resin (lane 4).

(H) *In vitro* pulldown assay showing that SUMO does not interact with H3K9me3Nucleosomes.

(I) Sample quality was checked by negative stain imaging.
