# Supplementary material for: The Chp1 chromodomain binds the H3K9me tail and the nucleosome core to assemble heterochromatin
Source: Cell Discov. 2016 Apr 19;2:16004–. doi: 10.1038/celldisc.2016.4 (PMC4849473; doi:10.1038/celldisc.2016.4)
Supplement: Supplementary Figure S2 [file celldisc20164-s2.pdf]

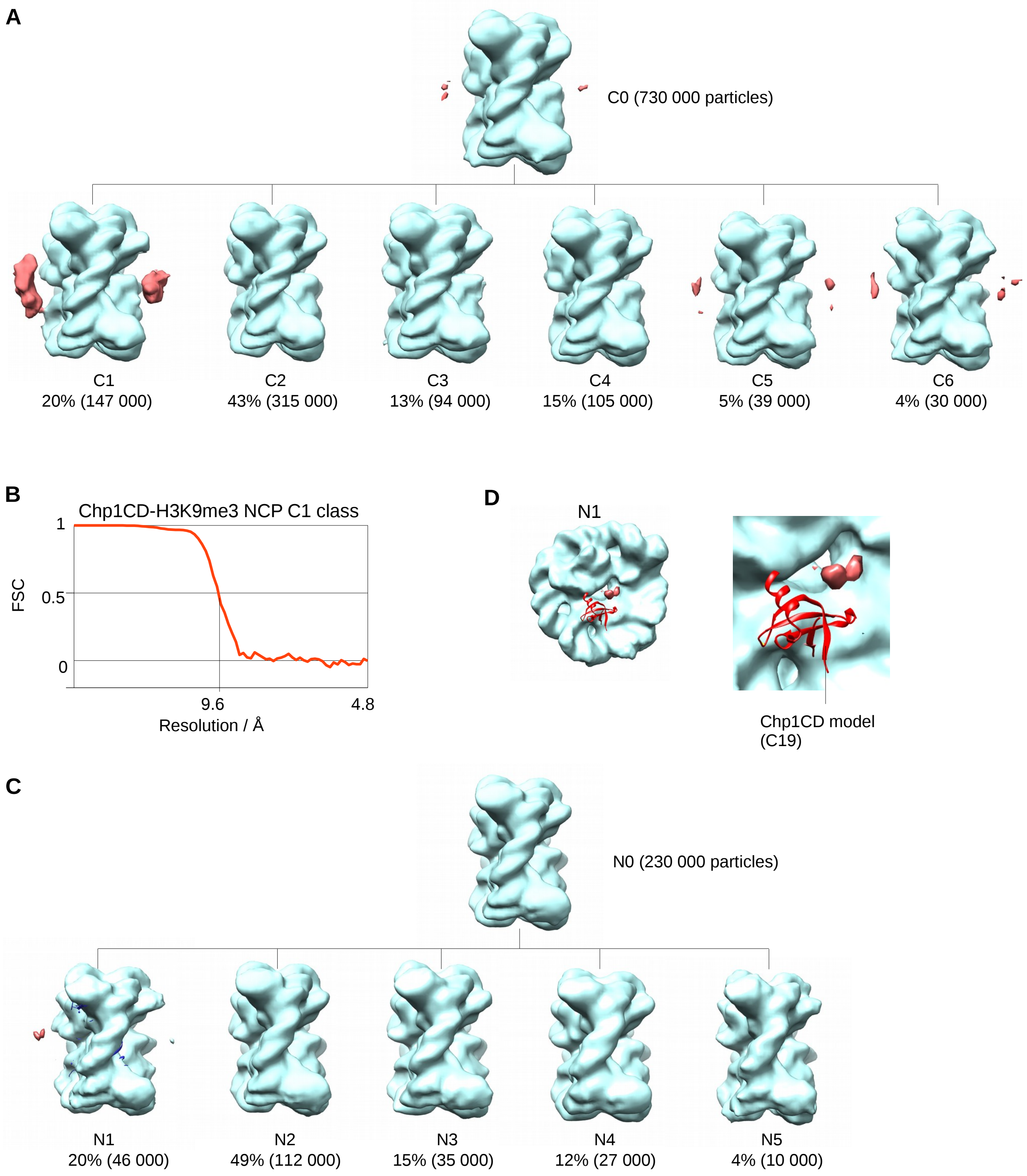
A
C0 (730 000 particles)
C1
20% (147 000)
C2
43% (315 000)
C3
13% (94 000)
C4
15% (105 000)
C5
5% (39 000)
C6
4% (30 000)
B
Chp1CD-H3K9me3 NCP C1 class
FSC
1
0.5
0
9.6
4.8
Resolution / Å
D
N1
Chp1CD model
(C19)
C
N0 (230 000 particles)
N1
20% (46 000)
N2
49% (112 000)
N3
15% (35 000)
N4
12% (27 000)
N5
4% (10 000)

Figure S2

Figure S2. Initial classification of Chp1CD-H3K9meNucleosome and H3K9meNucleosome datasets.

(A) Classification of the Chp1CD-H3K9meNucleosome complex. Class C1, containing 20% of all the particles, shows a strong density in addition to the core nucleosome particle. Classes C5 and C6 show a weak and noisy density in addition to the core nucleosome particle.

(B) Fourier shell correlation (FSC) curve showing the resolution of C1 cryo-EM maps. The resolution is shown at 0.5 cutoff. The mask including nucleosome and the Chp1 density was applied for resolution calculation.

(C) Classification of the H3K9meNucleosome control. No class contains a strong density in addition to the core nucleosome particle. Class N1 shows a weak and noisy density in addition to the core nucleosome particle similar to the classes C5 and C6 of the Chp1CD-H3K9meNucleosome complex. These densities might be generated by histone tails.

(D) Superposition of N1 cryo-EM map and Chp1CD model docked into C15 density (see later) shows no overlap between Chp1CD and the weak and noisy density in the N1 nucleosome map.
