# Supplementary material for: The Chp1 chromodomain binds the H3K9me tail and the nucleosome core to assemble heterochromatin
Source: Cell Discov. 2016 Apr 19;2:16004–. doi: 10.1038/celldisc.2016.4 (PMC4849473; doi:10.1038/celldisc.2016.4)
Supplement: Supplementary Figure S3 [file celldisc20164-s3.pdf]

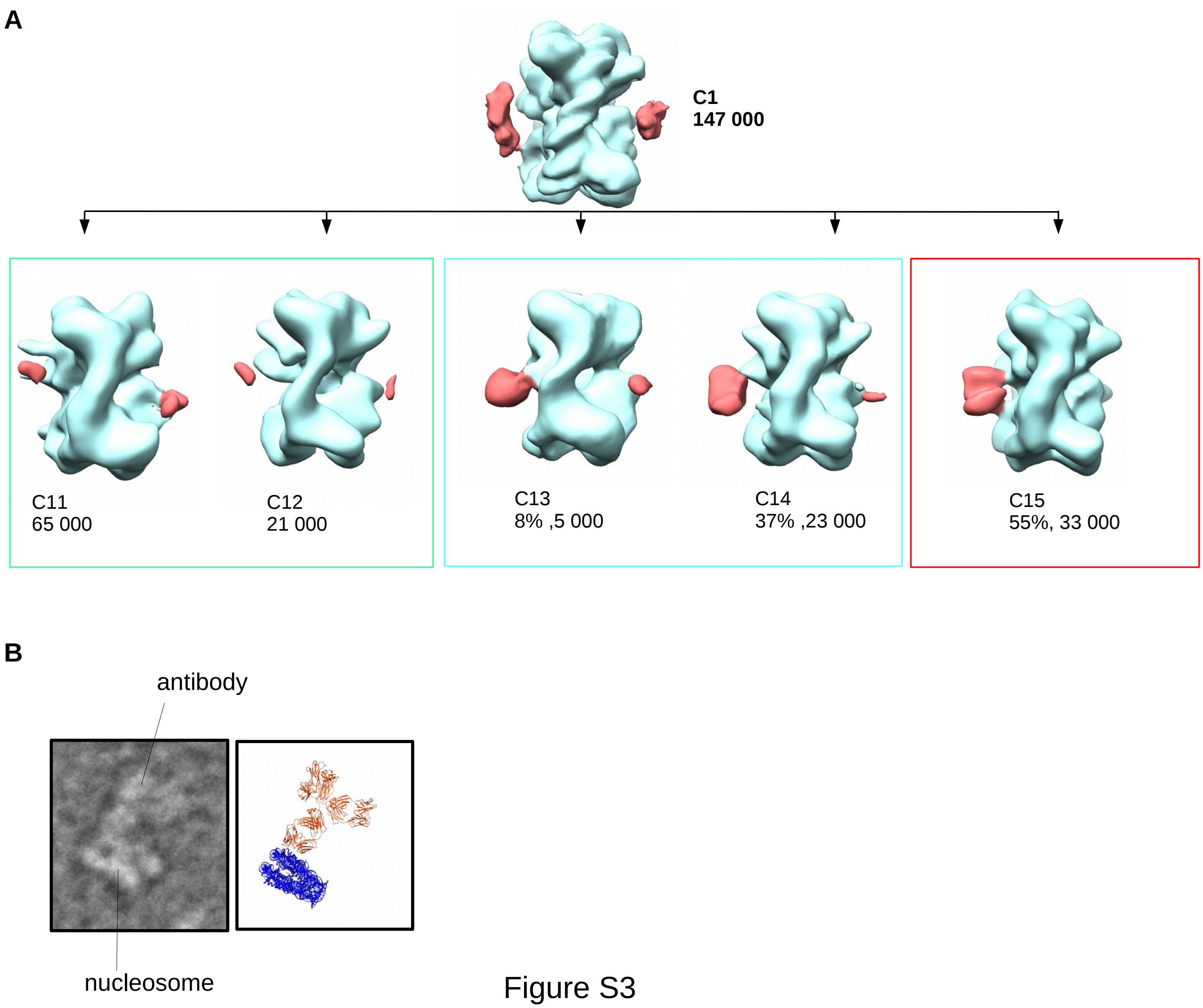

Figure S3

Figure S3. Classification of the Chp1CD-H3K9me3Nucleosome complex.

(A) Classification of the Chp1CD-H3K9me3Nucleosome complex class C1. 5 classes (C11-C15) were generated from C1 class particles. Each class contains a distinct density for Chp1CD associated with the nucleosome. Approximate number of particles (rounded to 1000) in each class is indicated below the image. Percentage of particles in each class is indicated. Classes C11 and C12, comprising 60% of particles, contain mainly empty nucleosomes (green box). 40% of particles (C13-C15) contain additional density close or attached to the nucleosome core. In classes C13 and C14 the additional density is less defined (light blue box). Class C15 has defined density attached to the nucleosome core (red box). All classes

are shown at the same contour level (0.00013) with exception of class C1 (0.00007).

(B) Representative negative stain image of antibody targeted Chp1CD-H3K9me3Nucleosome complex (left). Model of nucleosome and antibody in a similar orientation is shown on the right (PDB 3LZ0,1IGY). Nucleosome and antibody bound to the side of the nucleosome core are clearly resolved. Antibody targets similar region where Chp1CD binds in Chp1CD-H3K9me3Nucleosome complex map.
