# Supplementary material for: The Chp1 chromodomain binds the H3K9me tail and the nucleosome core to assemble heterochromatin
Source: Cell Discov. 2016 Apr 19;2:16004–. doi: 10.1038/celldisc.2016.4 (PMC4849473; doi:10.1038/celldisc.2016.4)
Supplement: Supplementary Figure S4 [file celldisc20164-s4.pdf]

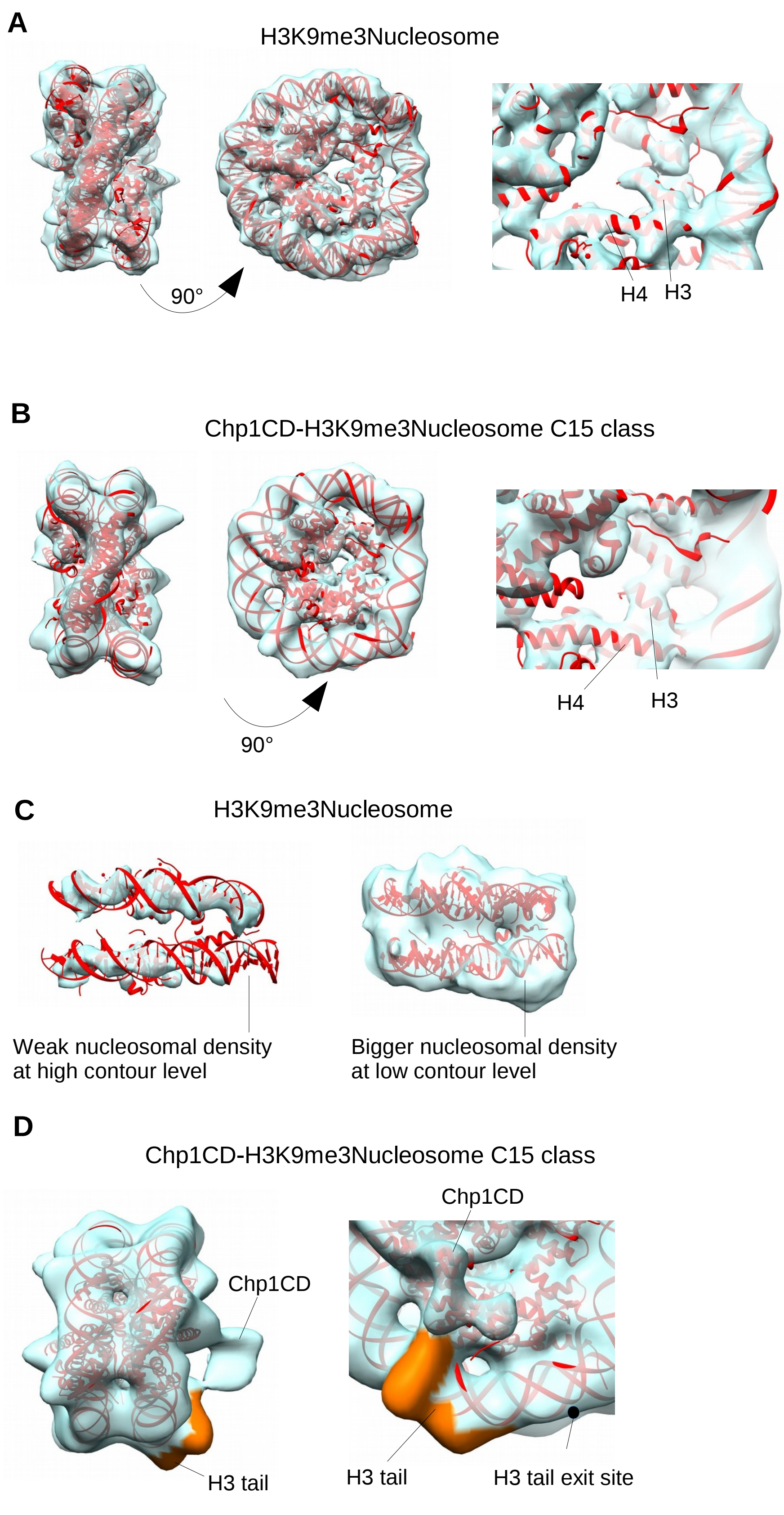
A
H3K9me3Nucleosome
90°
H4
H3
B
Chp1CD-H3K9me3Nucleosome C15 class
H4
H3
90°
C
H3K9me3Nucleosome
Weak nucleosomal density
at high contour level
Bigger nucleosomal density
at low contour level
D
Chp1CD-H3K9me3Nucleosome C15 class
Chp1CD
Chp1CD
H3 tail
H3 tail
H3 tail exit site

Figure S4

Figure S4. Fitting of the nucleosome and the Chp1CD crystal structures into cryo-EM density maps.

(A) Crystal structure of the nucleosome core particle (PDB 3LZ0) was unambiguously docked into the nucleosome cryo-EM map using Chimera auto-fit option. All α-helices are clearly resolved in the core of the nucleosome.

(B) Crystal structure of the nucleosome core particle (PDB 3LZ0) was unambiguously docked into the Chp1CD-H3K9me3Nucleosome complex cryo-EM map using Chimera auto-fit option. Many secondary structure elements (α-helices) are resolved in the core of the nucleosome.

(C) Nucleosome cryo EM map is shown at high contour level. Density for DNA at entry/exit site is not visible at high contour level. At low contour the DNA density at entry/exit site is larger and undefined. This indicates higher but restricted mobility of last 7-8 bp of DNA at entry/exit sites.

(D) Chp1CD-H3K9me3Nucleosome complex cryo-EM map shown at lower contour levels where the density can be traced from Chp1CD to the DNA and than along the DNA to the site where H3 exits the globular core of the nucleosome. This suggests that this density is generated by H3 tail which follows a somewhat defined path after interaction with Chp1CD.
