# Supplementary material for: The Chp1 chromodomain binds the H3K9me tail and the nucleosome core to assemble heterochromatin
Source: Cell Discov. 2016 Apr 19;2:16004–. doi: 10.1038/celldisc.2016.4 (PMC4849473; doi:10.1038/celldisc.2016.4)
Supplement: Supplementary Figure S5 [file celldisc20164-s5.pdf]

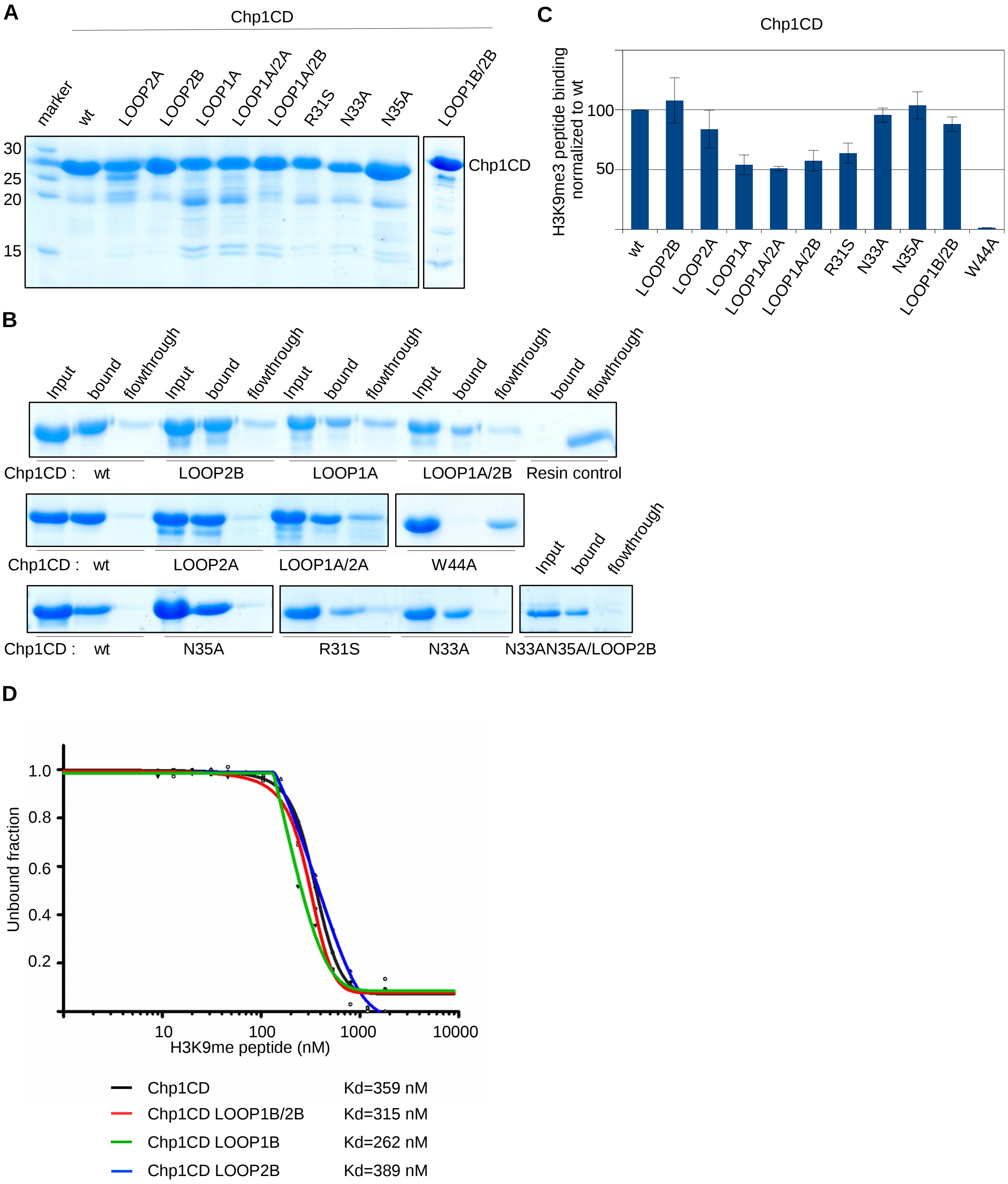
A
Chp1CD
marker
wt
LOOP2A
LOOP2B
LOOP1A
LOOP1A/2A
LOOP1A/2B
R31S
N33A
N35A
LOOP1B/2B
30
25
20
15
Chp1CD
C
Chp1CD
H3K9me3 peptide binding normalized to wt
100
50
wt
LOOP2B
LOOP2A
LOOP1A
LOOP1A/2A
LOOP1A/2B
R31S
N33A
N35A
LOOP1B/2B
W44A
B
Input
bound
flowthrough
Chp1CD : wt
LOOP2B
LOOP1A
LOOP1A/2B
Resin control
Chp1CD : wt
LOOP2A
LOOP1A/2A
W44A
Chp1CD : wt
N35A
R31S
N33A
N33AN35A/LOOP2B
D
Unbound fraction
1.0
0.8
0.6
0.4
0.2
10
100
1000
10000
H3K9me peptide (nM)
Chp1CD Kd=359 nM
Chp1CD LOOP1B/2B Kd=315 nM
Chp1CD LOOP1B Kd=262 nM
Chp1CD LOOP2B Kd=389 nM

Figure S5

Figure S5. Chp1CD LOOP1B and 2B mutations do not affect interaction with H3K9me tail.

(A) Coomassie stained SDS polyacrylamide gel showing purified Chp1CD mutants.

(B) Peptide binding assay showing the interaction of various Chp1CD mutants with the H3K9me3 peptide. Peptide binding of Chp1CD mutants is normalized to the wt binding in the same assay.

(C) Quantification of 3 independent peptide binding assays. The Chp1CD R31S and LOOP1 mutants show a defect in binding of the H3K9me peptide. LOOP2A/B, N33A, N35A and LOOP1B/LOOP2B had no defect in binding of the H3K9me peptide. Control Chp1CD W44A mutant has a complete loss in binding to H3K9me peptide.

(D) Thermophoresis assay showing binding curves of wild type Chp1CD and Chp1CD LOOP1B, LOOP2B and LOOP1B/2B mutants to H3K9me3 peptide. Kd is shown below the curve.
