# Supplementary material for: The Chp1 chromodomain binds the H3K9me tail and the nucleosome core to assemble heterochromatin
Source: Cell Discov. 2016 Apr 19;2:16004–. doi: 10.1038/celldisc.2016.4 (PMC4849473; doi:10.1038/celldisc.2016.4)
Supplement: Supplementary Figure S6 [file celldisc20164-s6.pdf]

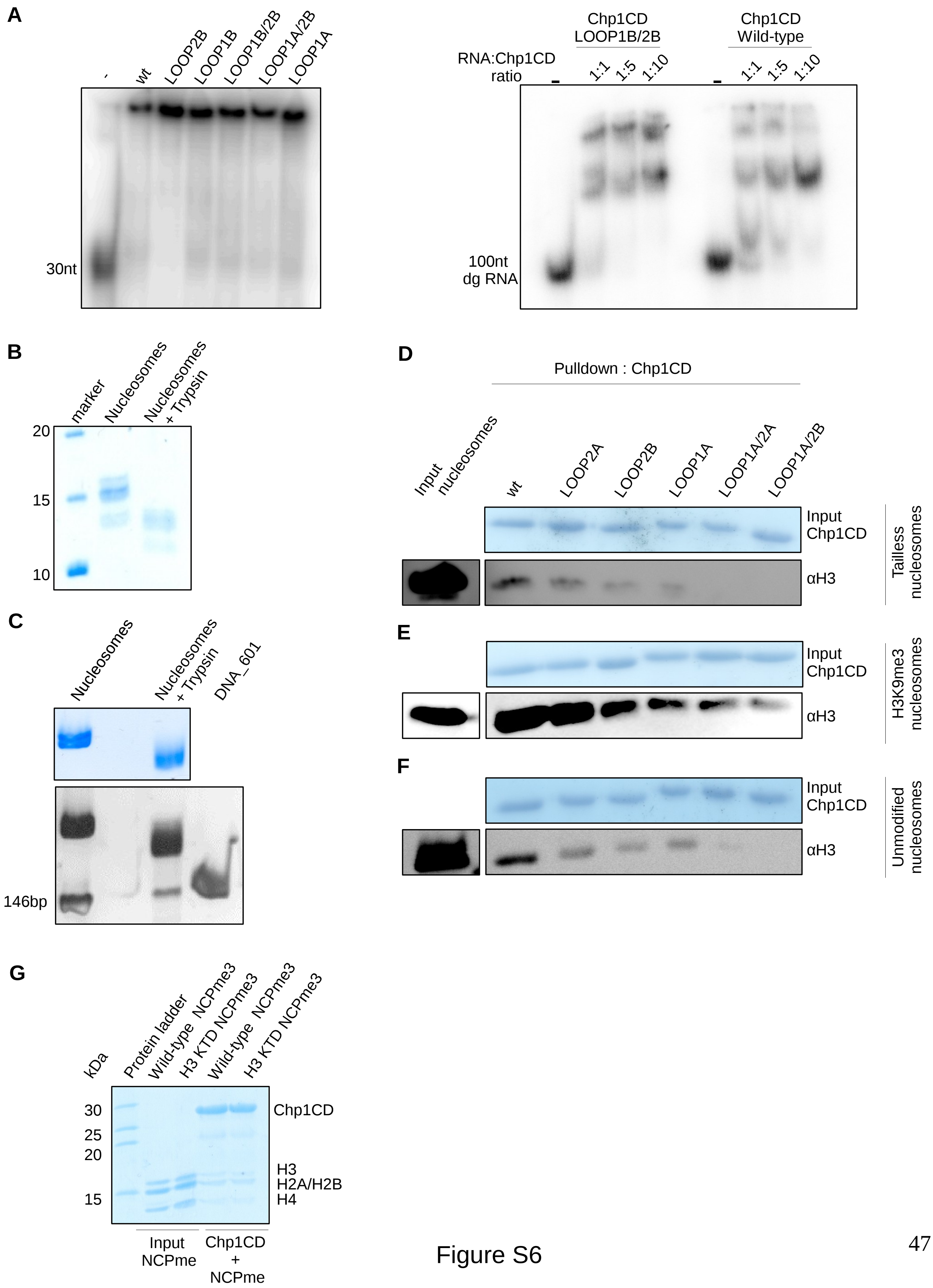
A
-
wt
LOOP2B
LOOP1B
LOOP1B/2B
LOOP1A/2B
LOOP1A
30nt
Chp1CD LOOP1B/2B
Chp1CD Wild-type
RNA:Chp1CD ratio
-
1:1
1:5
1:10
-
1:1
1:5
1:10
100nt dg RNA
B
marker
Nucleosomes
Nucleosomes + Trypsin
20
15
10
C
Nucleosomes
Nucleosomes + Trypsin
DNA_601
146bp
D
Pulldown : Chp1CD
Input nucleosomes
wt
LOOP2A
LOOP2B
LOOP1A
LOOP1A/2A
LOOP1A/2B
Input Chp1CD
αH3
Tailless nucleosomes
E
Input Chp1CD
αH3
H3K9me3 nucleosomes
F
Input Chp1CD
αH3
Unmodified nucleosomes
G
kDa
Protein ladder
Wild-type NCPme3
H3 KTD NCPme3
Wild-type NCPme3
H3 KTD NCPme3
30
25
20
15
Chp1CD
H3
H2A/H2B
H4
Input NCPme
Chp1CD + NCPme

Figure S6

**Figure S6.** Chp1CD interaction with the core of the nucleosome is abolished in Chp1CD loop mutants.

**(A)** Gel shift assay showing that wild type Chp1CD and LOOP1B and 2B mutants bind 30nt RNA (left) and 100nt RNA (right).

**(B)** SDS PAGE showing histones after tails were removed by trypsin.

**(C)** Native gel stained by coomassie and SybrGreen showing that nucleosomes are still intact after tails were removed by trypsin.

**(D)** *In vitro* pulldown assay showing that Chp1CD LOOP mutants abolish interaction with the core of the nucleosome.

**(E)** *In vitro* pulldown assay showing that Chp1CD LOOP mutants strongly reduce interaction with H3K9me3Nucleosomes.

**(F)** *In vitro* pulldown assay showing that Chp1CD LOOP mutants abolish interaction with non modified nucleosomes.

**(G)** *In vitro* pulldown assay showing that H3K79A/T80A/D81A mutations are not sufficient to abolish Chp1CD interaction with H3K9me3Nucleosomes.
