# Supplementary material for: The Chp1 chromodomain binds the H3K9me tail and the nucleosome core to assemble heterochromatin
Source: Cell Discov. 2016 Apr 19;2:16004–. doi: 10.1038/celldisc.2016.4 (PMC4849473; doi:10.1038/celldisc.2016.4)
Supplement: Supplementary Figure S7 [file celldisc20164-s7.pdf]

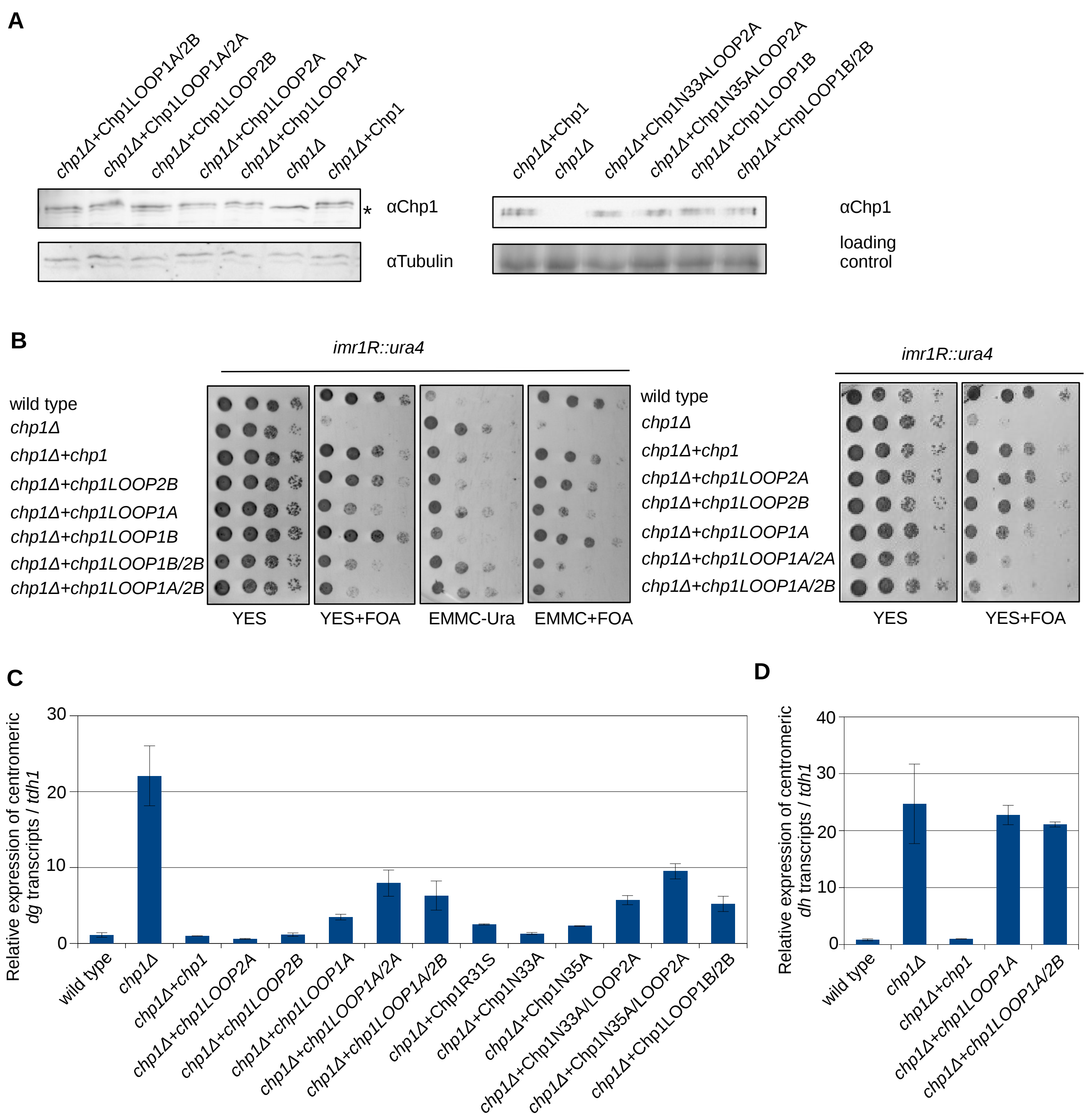

A
chp1Δ+Chp1LOOP1A/2B
chp1Δ+Chp1LOOP1A/2A
chp1Δ+Chp1LOOP2B
chp1Δ+Chp1LOOP2A
chp1Δ+Chp1LOOP1A
chp1Δ
chp1Δ+Chp1
*
αChp1
αTubulin
chp1Δ+Chp1
chp1Δ
chp1Δ+Chp1N33ALOOP2A
chp1Δ+Chp1N35ALOOP2A
chp1Δ+Chp1LOOP1B
chp1Δ+ChpLOOP1B/2B
αChp1
loading control
B
imr1R::ura4
wild type
chp1Δ
chp1Δ+chp1
chp1Δ+chp1LOOP2B
chp1Δ+chp1LOOP1A
chp1Δ+chp1LOOP1B
chp1Δ+chp1LOOP1B/2B
chp1Δ+chp1LOOP1A/2B
YES
YES+FOA
EMMC-Ura
EMMC+FOA
imr1R::ura4
wild type
chp1Δ
chp1Δ+chp1
chp1Δ+chp1LOOP2A
chp1Δ+chp1LOOP2B
chp1Δ+chp1LOOP1A
chp1Δ+chp1LOOP1A/2A
chp1Δ+chp1LOOP1A/2B
YES
YES+FOA
C
Relative expression of centromeric dg transcripts / tdh1
30
20
10
0
wild type
chp1Δ
chp1Δ+chp1
chp1Δ+chp1LOOP2A
chp1Δ+chp1LOOP2B
chp1Δ+chp1LOOP1A
chp1Δ+chp1LOOP1A/2A
chp1Δ+chp1LOOP1A/2B
chp1Δ+Chp1R31S
chp1Δ+Chp1N33A
chp1Δ+Chp1N35A
chp1Δ+Chp1N33A/LOOP2A
chp1Δ+Chp1N35A/LOOP2A
chp1Δ+Chp1LOOP1B/2B
D
Relative expression of centromeric dh transcripts / tdh1
40
30
20
10
0
wild type
chp1Δ
chp1Δ+chp1
chp1Δ+chp1LOOP1A
chp1Δ+chp1LOOP1A/2B

Figure S7

Figure S7. Mutations in Chp1 LOOP1 and LOOP2 lead to de-repression of pericentromeric transcripts.

(A) Western blot showing expression of mutated Chp1 reintroduced into *chp1Δ* cells. All mutant proteins are expressed at the same level compared to the tubulin or loading control (amido black stain of the membrane). * denotes an unspecific band than runs slightly lower than Chp1.

(B) Silencing assay showing that LOOP1/2 mutant have a defect in heterochromatin formation at centromeric repeats. 10-fold serial dilutions were plated.

(C) Relative expression of centromeric *dg* transcripts in wt and Chp1 mutant cells. Various Chp1 mutants are showing accumulation of pericentromeric *dg* transcripts. Even single mutations in loop1 (N35A) has an increase in *dg* transcripts. Accumulation of *dg* transcripts is much higher when N35 and N33 are combined with the LOOP2A/B mutants.

(D) Relative expression of centromeric *dh* transcripts in wt and Chp1 mutant cells. Various Chp1 mutants are showing accumulation of pericentromeric *dh* transcripts.
