# Supplementary material for: The Chp1 chromodomain binds the H3K9me tail and the nucleosome core to assemble heterochromatin
Source: Cell Discov. 2016 Apr 19;2:16004–. doi: 10.1038/celldisc.2016.4 (PMC4849473; doi:10.1038/celldisc.2016.4)
Supplement: Supplementary Figure S8 [file celldisc20164-s8.pdf]

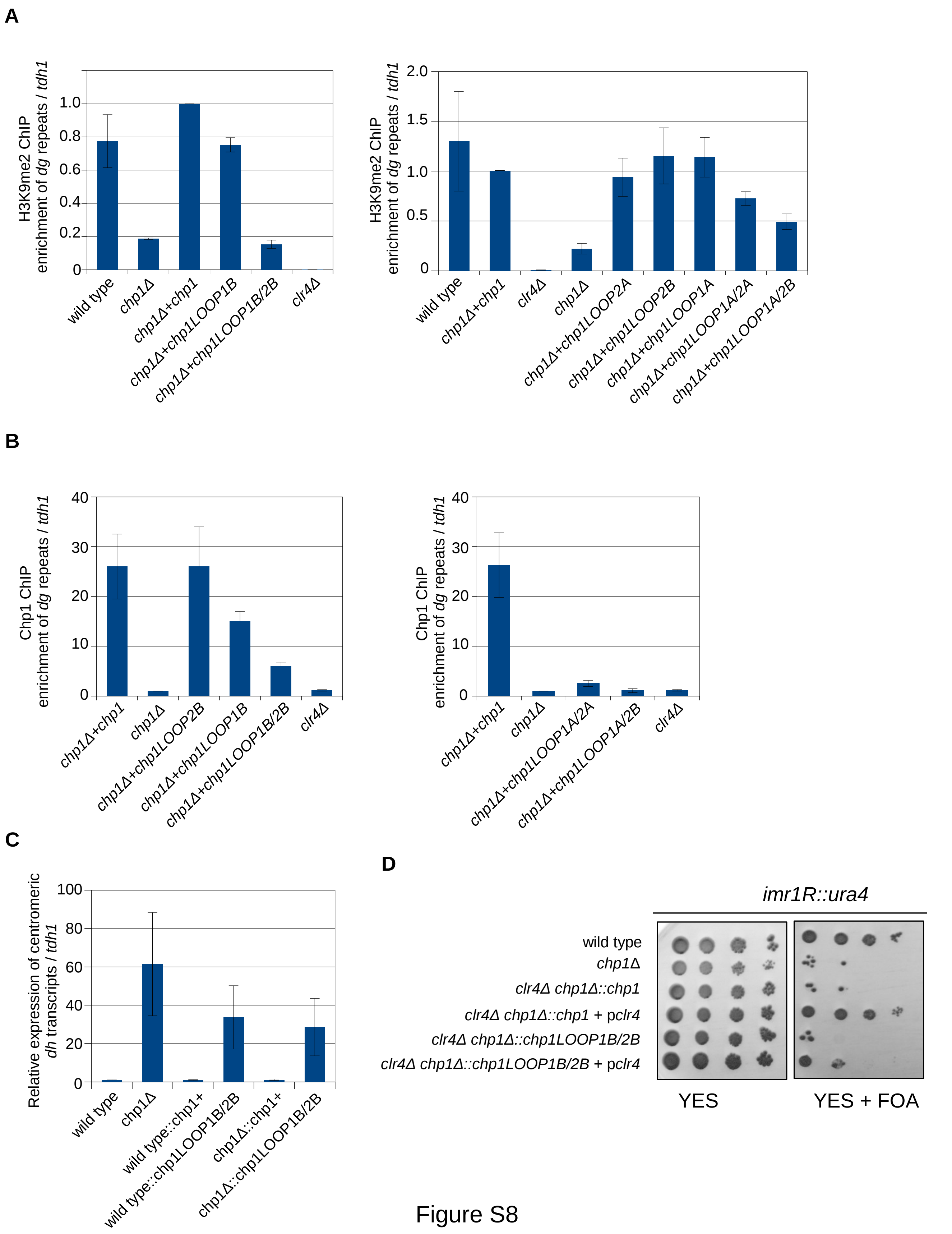
A
H3K9me2 ChIP enrichment of dg repeats / tdh1
wild type
chp1Δ
chp1Δ+chp1
chp1Δ+chp1LOOP1B
chp1Δ+chp1LOOP1B/2B
clr4Δ
clr4Δ
chp1Δ+chp1LOOP2A
chp1Δ+chp1LOOP2B
chp1Δ+chp1LOOP1A
chp1Δ+chp1LOOP1A/2A
chp1Δ+chp1LOOP1A/2B
B
Chp1 ChIP enrichment of dg repeats / tdh1
C
Relative expression of centromeric dh transcripts / tdh1
wild type::chp1+
wild type::chp1LOOP1B/2B
chp1Δ::chp1+
chp1Δ::chp1LOOP1B/2B
D
imr1R::ura4
clr4Δ chp1Δ::chp1
clr4Δ chp1Δ::chp1 + pclr4
clr4Δ chp1Δ::chp1LOOP1B/2B
clr4Δ chp1Δ::chp1LOOP1B/2B + pclr4
YES
YES + FOA

Figure S8

Figure S8. Mutations in Chp1 LOOP1 and LOOP2 lead to reduced localization of Chp1 and lower levels of H3K9me at pericentromeric repeats.

(A) ChIP experiment showing that H3K9me is reduced at centromeric *dg* repeats in Chp1CD LOOP1/2 mutant cells. Error bars indicate standard error mean.

(B) ChIP experiment showing that Chp1CD mutants are less efficiently recruited to centromeric *dg* repeats. Error bars indicate standard error mean.

(C) Relative expression of centromeric *dh* transcripts in wt and genomically integrated Chp1 mutant cells. Genomically integrated Chp1 mutants are showing accumulation of pericentromeric *dh* transcripts to the levels close to *chp1Δ* cells.

(D) Silencing assay showing that Chp1CDLOOP1B/2B mutant has a defect in heterochromatin establishment at centromeric repeats. 10-fold serial dilutions were plated.
