# Supplementary material for: The Chp1 chromodomain binds the H3K9me tail and the nucleosome core to assemble heterochromatin
Source: Cell Discov. 2016 Apr 19;2:16004–. doi: 10.1038/celldisc.2016.4 (PMC4849473; doi:10.1038/celldisc.2016.4)
Supplement: Supplementary Figure S9 [file celldisc20164-s9.pdf]

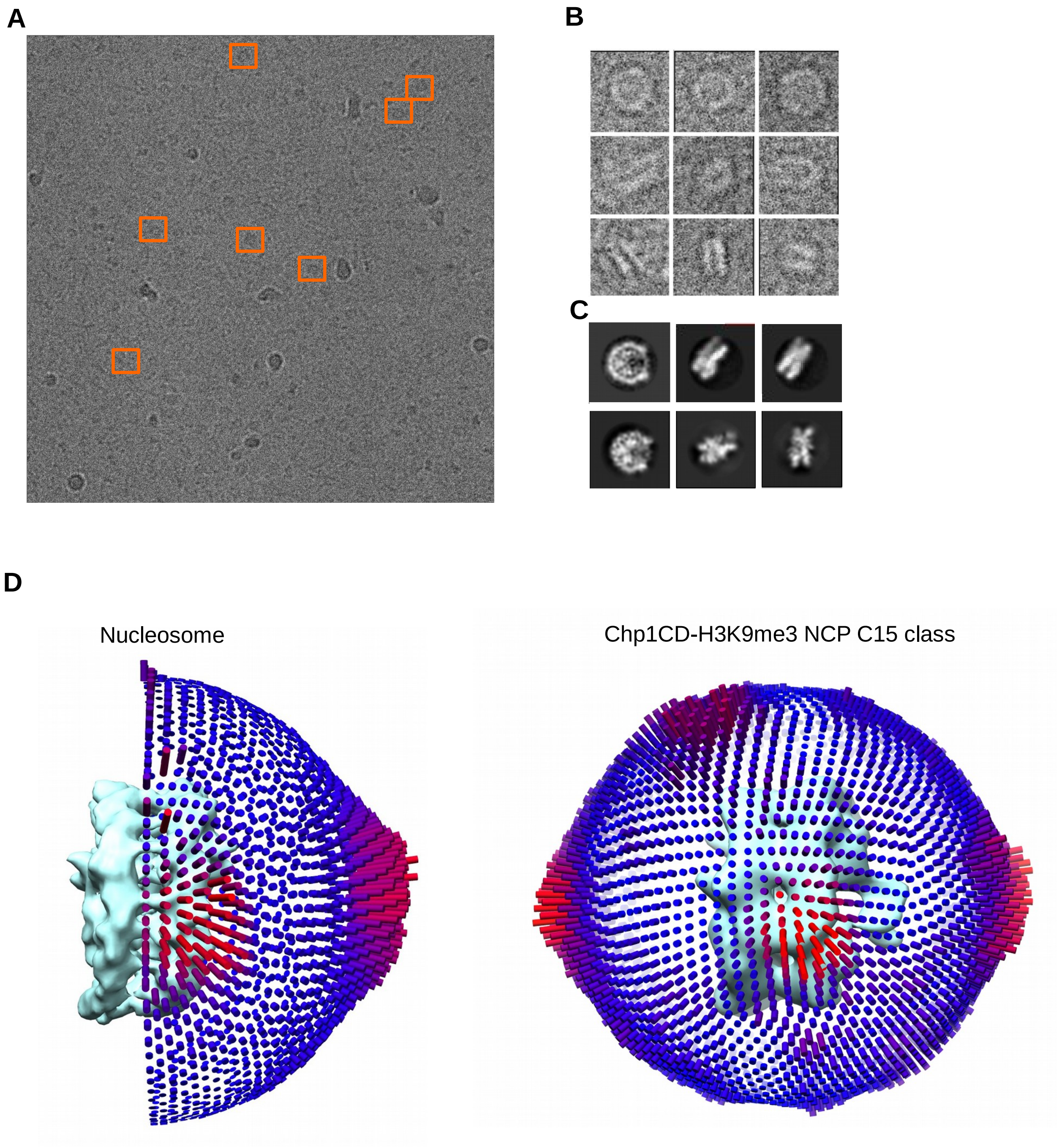

Figure S9

Figure S9. Cryo-EM data analysis supporting information.

(A) Representative cryo-EM raw micrograph collected with Titan Krios electron microscope at 200 keV. Several single particles are marked.

(B) Representative single particles showing nucleosomes in different orientations (side, tilted and top views).

(C) Representative 2D class averages showing nucleosomes in different orientations (side, tilted and top views) (top) and Chp1CD-H3K9meNucleosome complex cryo-EM map re-projections (bottom).

(D) Euler angle distribution for the final refinement step of nucleosome and Chp1CD-H3K9meNucleosome complex maps.
