# Supplementary material for: The Chp1 chromodomain binds the H3K9me tail and the nucleosome core to assemble heterochromatin
Source: Cell Discov. 2016 Apr 19;2:16004–. doi: 10.1038/celldisc.2016.4 (PMC4849473; doi:10.1038/celldisc.2016.4)
Supplement: Supplementary Table S1 [file celldisc20164-s10.pdf]

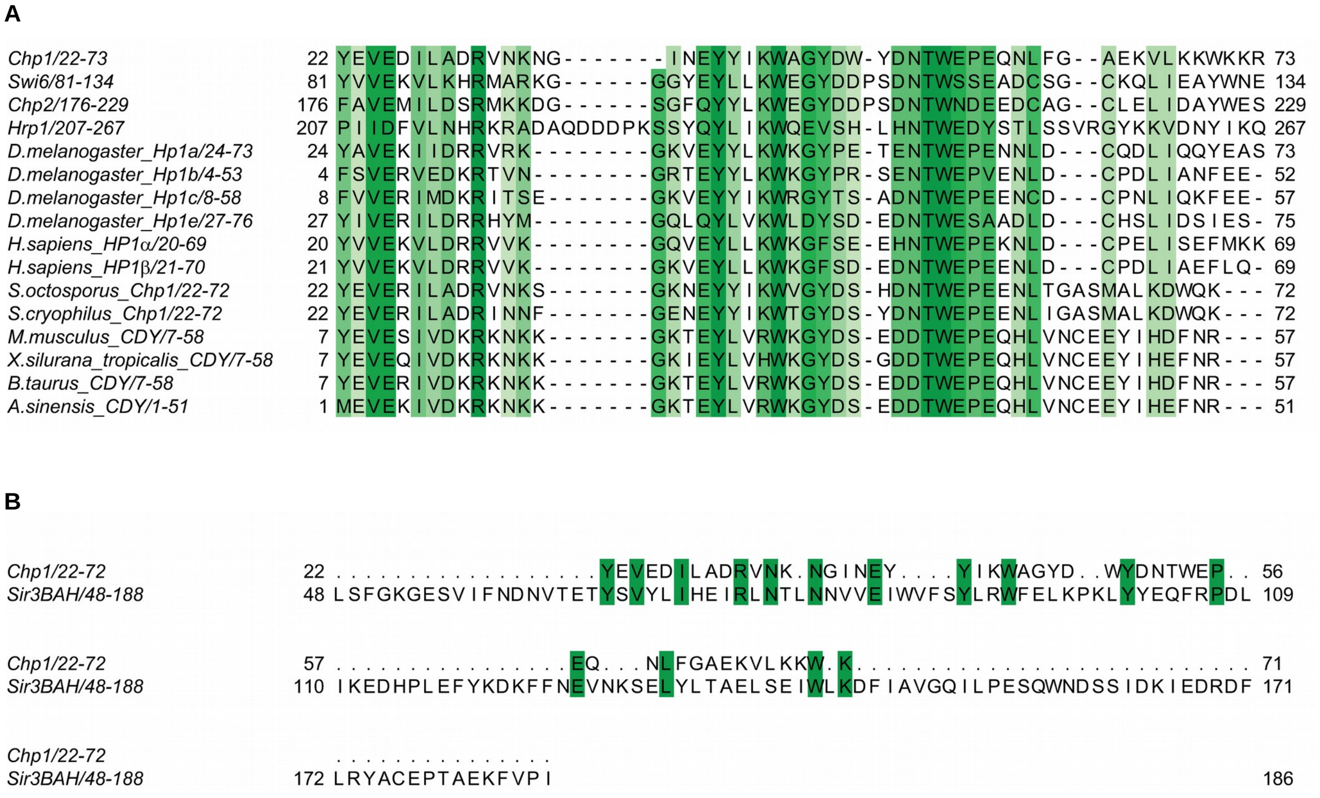

Table S1

Table S1. Sequence alignment of Chp1CD.

(A) Sequence alignment of fission yeast Chp1CD with chromodomains from fission yeast, Drosophila melanogaster (DM) and Homo Sapiens (HS). Conservation is shown in shades of green (dark green meaning highly conserved). R31, D51 and N52 are conserved in all chromodomains, while N33 is conserved in a subset of chromodomains.

(B) Sequence alignment of fission yeast Chp1CD with the Sir3BAH domain from *S. cerevisiae*. Conservation is shown in shades of green (dark green meaning highly conserved). The residues in loop1 which forms primary interaction with the nucleosome are conserved between Chp1CD and Sir3BAH.
