# Supplementary material for: The Chp1 chromodomain binds the H3K9me tail and the nucleosome core to assemble heterochromatin
Source: Cell Discov. 2016 Apr 19;2:16004–. doi: 10.1038/celldisc.2016.4 (PMC4849473; doi:10.1038/celldisc.2016.4)
Supplement: Supplementary Table S2 [file celldisc20164-s11.pdf]

OLIGONUCLEOTIDES	SEQUENCE
**SphI-BamHI chp1+ ORF + endogenous promoter cloning**	
346F	CACGCATGCGCAATTATCAAAAGAGGTCGTG
174R	CACGGATCCCTATTTTAAACCAATAGCTCTCATAAAAGT
**Chp1 Chromodomain mutants**	
349F.R31S	CTGACTCGGTAAATAAAAATGGC
349R(common to R31S, N33A, N35A, LOOP1)	CAAGGATATCTTCCACCTC
351F.N33A	CTGACCGCGTAGCTAAAAATGGC
358F.N35A	CTGACCGCGTAAATAAAGCTGGCATAAAC
359F.E39S	AAACTCGTATTATATAAAATGGGCTGGCTAT
359R.E39S	ATGCCATTTTTATTTACGCGGTC
354F.LOOP1(R31S,N33A,N35A)	CTGACTCGGTAGCTAAAGCTGGCATAAACGAATAT
356F.LOOP2A(W49Y50D51/AAA)	GGCTATGATGCTGCTGCTAATACTTGGGAACCTGAACAAA
356R.LOOP2A/2B	AGCCCATTTTATATAATATTCGTTTATG
357F.LOOP2B(N52A)	GGCTATGATTGGTATGATGCTACTTGGGAACCT
442F.LOOP1 double mutant(N33A,N35A)	CTGACCGCGTAGCTAAAGCTGGCATAAACGAATAT
**RT-qPCR and ChIP oligonucleotides**	
110A.TDH1_F	CCAAGCCTACCAACTACG
110A.TDH1_R	AGAGACGAGCTTGACGAA
110F.DGF_F	CTGCGGTTCACCCTTAACAT
110F.DGF_R	CAACTGCGGATGGAAAAAGT

Table S2. List of oligonucleotides used in this study.
