# Supplementary material for: The Chp1 chromodomain binds the H3K9me tail and the nucleosome core to assemble heterochromatin
Source: Cell Discov. 2016 Apr 19;2:16004–. doi: 10.1038/celldisc.2016.4 (PMC4849473; doi:10.1038/celldisc.2016.4)
Supplement: Supplementary Table S3 [file celldisc20164-s12.pdf]

plasmid

p463	pET28a - 6His SUMO Chp1 Chromo-Domain (residues 15-75)
p515	pREP1 - chp1 promoter_chp1_nmt1 terminator
p522	pREP1 - chp1 promoter_chp1 R31S_nmt1 terminator
p523	pREP1 - chp1 promoter_chp1 N33A_nmt1 terminator
p524	pREP1 - chp1 promoter_chp1 E39S_nmt1 terminator
p525	pREP1 - chp1 promoter_chp1 R31SN33AN35A_nmt1 terminator
p526	pREP1 - chp1 promoter_chp1 N52A_nmt1 terminator
p527	pREP1 - chp1 promoter_chp1 W49AY50AD51A_nmt1 terminator
p545	pREP1 - chp1 promoter_chp1 N52A/ R31SN33AN35A _nmt1 terminator
p546	pREP1 - chp1 promoter_chp1 W49AY50AD51A/ R31SN33AN35A_nmt1 terminator
p557	pET28a - 6His SUMO Chp1 Chromo-Domain N52A
p558	pET28a - 6His SUMO Chp1 Chromo-Domain W49AY50AD51A
p564	pREP1 - chp1 promoter_chp1 N35A_nmt1 terminator
p569	pET28a - 6His SUMO Chp1 Chromo-Domain W49AY50AD51A/ R31SN33AN35A
p571	pET28a - 6His SUMO Chp1 Chromo-Domain R31SN33AN35A
p576	pET28a - 6His SUMO Chp1 Chromo-Domain R31SN33AN35A/N52A
p597	pET28a - 6His SUMO Chp1 Chromo-Domain R31S
p599	pET28a - 6His SUMO Chp1 Chromo-Domain N35A
p601	pET28a - 6His SUMO Chp1 Chromo-Domain E39S
p606	pET28a - 6His SUMO Chp1 Chromo-Domain N33A

p638	pREP1 - chp1 promoter_chp1 N33AN35A_nmt1 terminator
p639	pREP1 - chp1 promoter_chp1 N33AN35A/W49AY50AD51A_nmt1 terminator
p406	pET3- *Xenopus laevis* histone H2A
p407	pET3- *Xenopus laevis* histone H2B
p408	pET3- *Xenopus laevis* histone H3
p409	pET3- *Xenopus laevis* histone H4
p420	pUC57 – 8 X 145bp 601 sequence (plasmid construct received from Davey CA and published in (Vasudevan et al., 2010))
p794	pREP1-(SphI) Region at the 5´ of Chp1 gene (Chromosome I, 2215500 – 2215055) (AscI) – HphMX6 resistance cassette –(SphI) Chp1 endogenous promoter (Chromosome I, 2214829 – 2214664) – *chp1+* ORF– (BamHI) Chp1 terminator (Chromosome I, 2210976 – 2210582) (BamHI).
p795	pREP1-(SphI) Region at the 5´ of Chp1 gene (Chromosome I, 2215500 – 2215055) (AscI) – HphMX6 resistance cassette –(SphI) Chp1 endogenous promoter (Chromosome I, 2214829 – 2214664) – *chp1LOOP1B/2B* ORF– (BamHI) Chp1 terminator (Chromosome I, 2210976 – 2210582) (BamHI).

Table S3. List of plasmids used in this study.
