# Supplementary material for: The Chp1 chromodomain binds the H3K9me tail and the nucleosome core to assemble heterochromatin
Source: Cell Discov. 2016 Apr 19;2:16004–. doi: 10.1038/celldisc.2016.4 (PMC4849473; doi:10.1038/celldisc.2016.4)
Supplement: Supplementary Table S4 [file celldisc20164-s13.pdf]

SP170	*h+ leu1-32 ura4-D18 imr1R(NCol)::ura4+ oriI ade6-216 Δchp1::TAP-KanMX6*
SP170 + p515	*h+ leu1-32 ura4-D18 imr1R(NCol)::ura4+ oriI ade6-216 Δchp1::TAP-KanMX6 + chp1+*
SP170 + p522	*h+ leu1-32 ura4-D18 imr1R(NCol)::ura4+ oriI ade6-216 Δchp1::TAP-KanMX6 + chp1R31S*
SP170 + p523	*h+ leu1-32 ura4-D18 imr1R(NCol)::ura4+ oriI ade6-216 Δchp1::TAP-KanMX6 + chp1N33A*
SP170 + p524	*h+ leu1-32 ura4-D18 imr1R(NCol)::ura4+ oriI ade6-216 Δchp1::TAP-KanMX6 + chp1E39S*
SP170 + p525	*h+ leu1-32 ura4-D18 imr1R(NCol)::ura4+ oriI ade6-216 Δchp1::TAP-KanMX6+ chp1R31SN33AN35A*
SP170 + p526	*h+ leu1-32 ura4-D18 imr1R(NCol)::ura4+ oriI ade6-216 Δchp1::TAP-KanMX6+ chp1N52A*
SP170 + p527	*h+ leu1-32 ura4-D18 imr1R(NCol)::ura4+ oriI ade6-216 Δchp1::TAP-KanMX6+ chp1 W49AY50AD51A*
SP170 + p545	*h+ leu1-32 ura4-D18 imr1R(NCol)::ura4+ oriI ade6-216 Δchp1::TAP-KanMX6+ chp1N52A/ R31SN33AN35A*
SP170 + p546	*h+ leu1-32 ura4-D18 imr1R(NCol)::ura4+ oriI ade6-216 Δchp1::TAP-KanMX6 + chp1 W49AY50AD51A/ R31SN33AN35A*
SP170 + p564	*h+ leu1-32 ura4-D18 imr1R(NCol)::ura4+ oriI ade6-216 Δchp1::TAP-KanMX6+ chp1N35A*
SP170 + p638	*h+ leu1-32 ura4-D18 imr1R(NCol)::ura4+ oriI ade6-216 Δchp1::TAP-KanMX6 + chp1 N33AN35A*
SP170 + p639	*h+ leu1-32 ura4-D18 imr1R(NCol)::ura4+ oriI ade6-216 Δchp1::TAP-KanMX6 + chp1 N33AN35A/W49AY50AD51A*

SP101	*h+ leu1-32 ura4-D18 imr1R(NCol)::ura4+ oriI ade6-216*
SP64	*h+ leu1-32 ura4-D18 imr1R(NCol)::ura4+ oriI ade6-216 Δclr4::KanMX6*
SP967	*h+ leu1-32 ura4-D18 imr1R(NCol)::ura4+ oriI ade6-216 chp1+:: HphMX6- chp1+(ChrI- 2214829- 2210582)*
SP968	*h+ leu1-32 ura4-D18 imr1R(NCol)::ura4+ oriI ade6-216 chp1+:: HphMX6- chp1LOOP1B/2B (ChrI- 2214829- 2210582)*
SP970	*h+ leu1-32 ura4-D18 imr1R(NCol)::ura4+ oriI ade6-216 Δchp1:: HphMX6- chp1+(ChrI- 2214829- 2210582)*
SP972	*h+ leu1-32 ura4-D18 imr1R(NCol)::ura4+ oriI ade6-216 Δchp1:: HphMX6- chp1LOOP1B/2B(ChrI- 2214829- 2210582)*
SP974	*h+ leu1-32 ura4-D18 imr1R(NCol)::ura4+ oriI ade6-216 chp1+:: HphMX6- chp1+(ChrI- 2214829- 2210582) Δclr4::KanMX6*
SP975	*h+ leu1-32 ura4-D18 imr1R(NCol)::ura4+ oriI ade6-216 chp1+:: HphMX6- chp1LOOP1B/2B (ChrI- 2214829- 2210582) Δclr4::KanMX6*

Table S4. List of *S. pombe* strains used in this study.
